# Rescue of ATXN3 neuronal toxicity in *Caenorhabditis* *elegans* by chemical modification of endoplasmic reticulum stress

**DOI:** 10.1242/dmm.029736

**Published:** 2017-12-01

**Authors:** Yasmin Fardghassemi, Arnaud Tauffenberger, Sarah Gosselin, J. Alex Parker

**Affiliations:** 1 Centre de recherche du Centre hospitalier de l'Université de Montréal (CRCHUM) Montréal, Québec H2X 0A9, Canada; 2 Département de biochimie et médecine moléculaire, Université de Montréal, Montréal, Québec H3T 1J4, Canada; 3 Département de pathologie et biologie cellulaire, Université de Montréal, Montréal, Québec H3T 1J4, Canada; 4 Département de neurosciences, Université de Montréal, Montréal, Québec H3T 1J4, Canada

**Keywords:** Ataxin 3, *Caenorhabditiselegans*, Endoplasmic reticulum stress, Guanabenz, Polyglutamine

## Abstract

Polyglutamine expansion diseases are a group of hereditary neurodegenerative disorders that develop when a CAG repeat in the causative genes is unstably expanded above a certain threshold. The expansion of trinucleotide CAG repeats causes hereditary adult-onset neurodegenerative disorders, such as Huntington's disease, dentatorubral–pallidoluysian atrophy, spinobulbar muscular atrophy and multiple forms of spinocerebellar ataxia (SCA). The most common dominantly inherited SCA is the type 3 (SCA3), also known as Machado–Joseph disease (MJD), which is an autosomal dominant, progressive neurological disorder. The gene causatively associated with MJD is *ATXN3*. Recent studies have shown that this gene modulates endoplasmic reticulum (ER) stress. We generated transgenic *Caenorhabditis* *elegans* strains expressing human *ATXN3* genes in motoneurons, and animals expressing mutant *ATXN3-CAG89* alleles showed decreased lifespan, impaired movement, and rates of neurodegeneration greater than wild-type *ATXN3-CAG10* controls. We tested three neuroprotective compounds (Methylene Blue, guanabenz and salubrinal) believed to modulate ER stress and observed that these molecules rescued *ATXN3-CAG89* phenotypes. Furthermore, these compounds required specific branches of the ER unfolded protein response (UPR^ER^), reduced global ER and oxidative stress, and polyglutamine aggregation. We introduce new *C. elegans* models for MJD based on the expression of full-length *ATXN3* in a limited number of neurons. Using these models, we discovered that chemical modulation of the UPR^ER^ reduced neurodegeneration and warrants investigation in mammalian models of MJD.

## Abstract (precis)

**Summary:** We introduce a novel *C. elegans* model for Machado–Joseph disease for use in preclinical drug discovery and identified guanabenz as a potent neuroprotective molecule.

## INTRODUCTION

Polyglutamine (poly-Q) expansion diseases are a class of dominantly inherited neurodegenerative disorders that develop when there is an abnormal expansion, and subsequent translation, of trinucleotide CAG repeats (Gatchel and Zoghbi, 2005; Matos et al., 2011; Shao and Diamond, 2007; Xu et al., 2015). These diseases are characterized by a selective loss of neurons along with physical and psychological complications (Matos et al., 2011). Indeed, the abnormal expansion of polyglutamine induces numerous pathological changes in patients, including modifications of the proteome leading to functional alterations, generation of toxic poly-Q protein species, protein aggregation, transcriptional dysregulation, proteotoxic stress and mitochondrial dysfunction (Shao and Diamond, 2007). However, the exact mechanism of disease pathogenesis is still not well understood. The poly-Q expansion diseases include several neurodegenerative disorders, such as Huntington's disease, dentatorubral–pallidoluysian atrophy, spinobulbar muscular atrophy and six forms of spinocerebellar ataxia (SCA) (Matos et al., 2011; Teixeira-Castro et al., 2015). SCAs are considered to be rare disorders, with a prevalence ranging from 0.3 to 2.0 per 100,000 (van de Warrenburg et al., 2002). SCA3 (spinocerebellar ataxia type 3), also known as Machado–Joseph disease (MJD), is considered to be the most common form of SCA worldwide (Schöls et al., 2004).

MJD is an autosomal dominant progressive neurological disorder characterized principally by ataxia, spasticity, peripheral neuropathy and ocular movement abnormalities (França et al., 2008). This disease is accompanied by neurodegeneration in selective regions, mainly in the cerebellum, basal ganglia, brainstem and spinal cord (Teixeira-Castro et al., 2011, 2015; Matos et al., 2011). Regarding brain function, it has been shown that metabolism is decreased in several regions of the nervous system, such as the cerebellum, brainstem and cerebral cortex, along with negative perturbations in both dopaminergic and cholinergic neurotransmission (Soon et al., 1997; Wüllner et al., 2005; Yen et al., 2000; Riess et al., 2008; Rub et al., 2008). MJD, constituting the most prevalent subtype of SCA, is more frequently observed among people of Portuguese/Azorean ancestry, with the highest prevalence in the Azorean island of Flores (1/239; Bettencourt et al., 2008). The gene causatively associated with MJD is *ATXN3* (ataxin 3) and is located on chromosome 14 (14q24.3-14q32.45; Kawaguchi et al., 1994; Takiyama et al., 1994). This gene encodes a poly-Q-containing protein named ataxin 3 (Kawaguchi et al., 1994).

Ataxin 3 has 339 amino acid residues, with an estimated molecular weight of 42 kDa for normal individuals (Kawaguchi et al., 1994). Healthy individuals have between 10-51 CAG repeats, which is expanded to 55-87 repeats in the disease state (Maciel et al., 2001; Cummings and Zoghbi, 2000). Ataxin 3 has several functional domains, including the N-terminus of the catalytic Josephin domain that presents a globular and a very conserved structure, followed by two ubiquitin interacting motifs (UIM) that are also considered as conserved regions, the poly-Q domain and finally, depending on the protein isoform, a third atypical UIM in the C-terminus tail (Goto et al., 1997; Li et al., 2015).

To aid the study of MJD, we turned to the model organism *Caenorhabditis elegans*. This nematode is 1 mm long, easy to maintain in laboratory settings, highly amenable to genetic manipulation, and is especially well suited for neuroscience research because of its comprehensively detailed neuronal lineage and interconnectivity of synapses that resembles aspects of the vertebrate nervous system (Stiernagle, 2006). Additionally, the *C. elegans* genome contains an orthologue of *ATXN3*, named *atx-3*. The *C. elegans* orthologue of human ataxin 3 is localized in both the nucleus and the cytoplasm, with higher levels observed in the cytoplasm. It has been shown that loss of *atx-3* activity results in changes in the expression of genes involved in several different pathways: the ubiquitin–proteasome pathway, signal transduction and cell structure and motility (Rodrigues et al., 2007; Kawaguchi et al., 1994). Ataxin 3 has been identified as participating in the endoplasmic reticulum network (Matos et al., 2011; Echtermeyer et al., 2011; Reina et al., 2010). We previously showed that several compounds, including Methylene Blue, salubrinal and guanabenz, target the endoplasmic reticulum (ER) stress response and protect against proteotoxicity in simple models of amyotrophic lateral sclerosis (ALS; Vaccaro et al., 2013, 2012a) and have beneficial effects in models of the neurological disorder hereditary spastic paraplegia (Julien et al., 2016). In this study, using our transgenic *C. elegans ATXN3* models, we explored whether small molecules that regulate ER stress response activity were able to rescue locomotor phenotypes, neuronal loss and the increased oxidative and ER stress observed in mutant transgenic animals as an early effort for MJD therapy development.

## RESULTS

### Expression of full-length human *ATXN3* in *C. elegans*

**Fig. 1.**
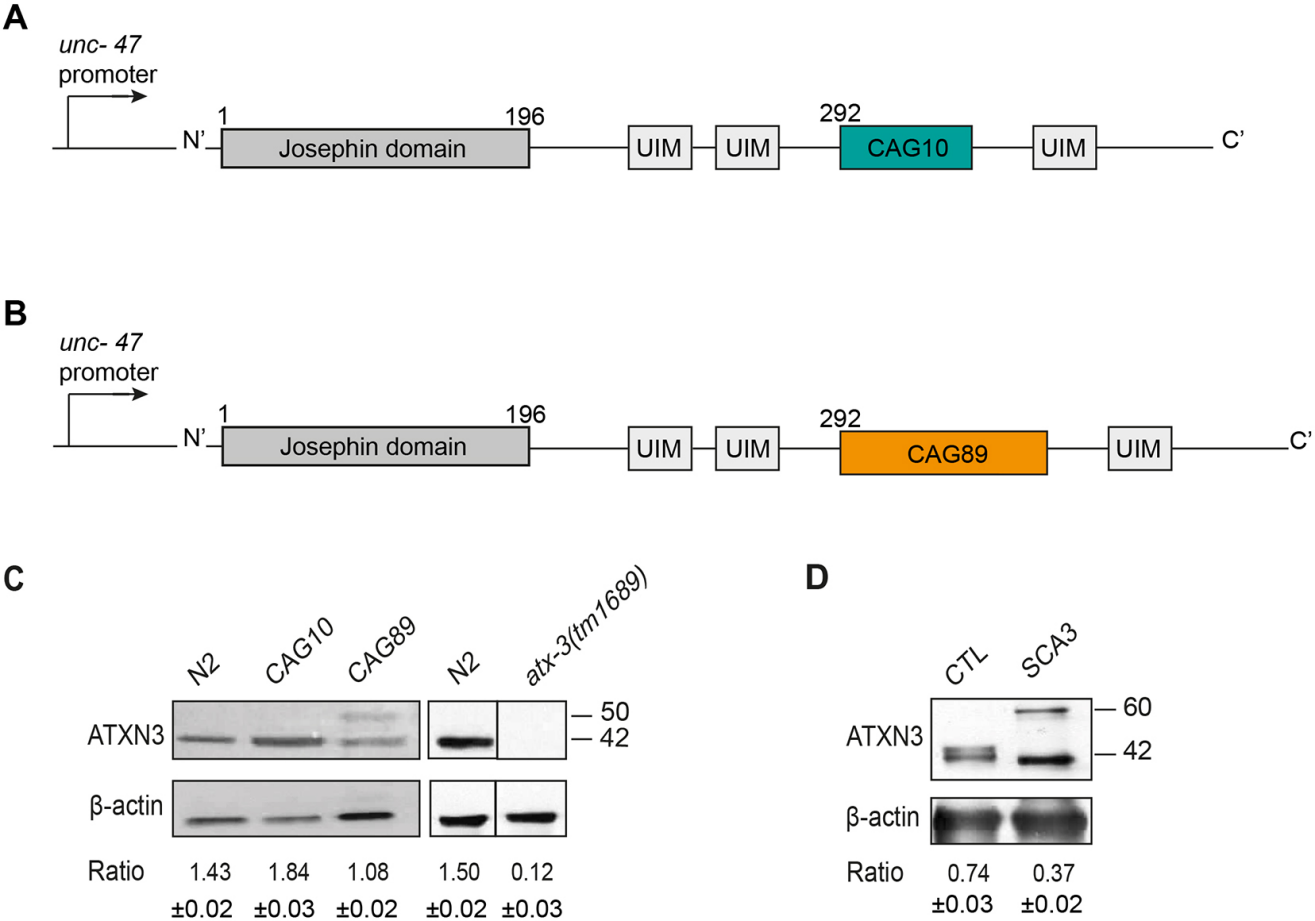
**Transgenic *C. elegansATXN3* model.** (A) Full-length wild-type human *ATXN3* containing 10 CAG trinucleotide repeat expansion or (B) full-length human ATXN3 with a polyglutamine expansion (CAG89) were expressed under the control of a *unc-47* promoter. CAG10/CAG89, polyglutamine sequences; UIM, interaction domains with ubiquitin. (C) Total protein levels for N2, *atx-3(tm1689)* and transgenic worms expressing *ATXN3-CAG10* or mutant *ATXN3-CAG89*. Antibody detection revealed signals for transgenic and N2 wild-type worms, but no corresponding signal was detected in *atx-3(tm1689)* samples. Bands observed at ∼42 kDa in size corresponding to full-length ATXN3 were observed in extracts from transgenic *ATXN3-CAG10* worms, and a slightly larger band at ∼50 kDa in size was observed for *ATXN3-CAG89* samples. A band was observed in non-transgenic N2 worms, probably representing the endogenous ATX-3 protein. No band was observed in the *atx-3(tm1689)* deletion mutant. The *atx-3(tm1689)* western blots were done independently from the transgenic *ATXN3* experiments. For the *ATXN3-CAG89* lane, the ratio of ATXN3 to actin was made using the top band. (D) Total protein levels from cells derived from healthy (control; CTL) and MJD (SCA3) patients. Staining showed signals for both healthy and MJD patients, having an estimated molecular weight of 42 kDa and 60 kDa, respectively. For all experiments, actin staining was used as a loading control, and the expression ratio ±s.e.m. of ATXN3 to actin was determined from three independent experiments. Representative western blots are shown.

Using Mos1-mediated single copy insertion (MoSCi) transposon-mediated single copy insertion into the genome (Frokjaer-Jensen et al., 2014), we created strains to model MJD by expressing full-length, human *ATXN3* in *C. elegans*. It has been shown previously that expressing ataxin 3 with 89 CAG repeats (CAG89) results in neurodegeneration, and in protein misfolding phenotypes in cell culture and *Drosophila* models (Stochmanski et al., 2012). Based on our previous neurodegeneration models (ALS models), we expressed human *ATXN3*, either wild-type *ATXN3-CAG10* (Fig. 1A) or mutant *ATXN3-CAG89* (Fig. 1B), in the worm's 26 GABAergic motoneurons using the promoter for the gene *unc-47*, which encodes a vesicular GABA transporter (Vaccaro et al., 2012a). In our experience, the *unc-47* modelling approach produces animals with strong phenotypes resulting from the expression of disease-associated proteins in a small number of neurons. Thus, in the context of chemical suppressor screens, relatively few neurons need to be exposed to small molecules to detect reversion of phenotypes.

We confirmed the expression of full-length human *ATXN3* in transgenic *C. elegans* strains by western blotting with a human specific anti-ATXN3 antibody. A band corresponding to wild-type *ATXN3-CAG10* and a larger band for the MJD-associated mutant *ATXN3-CAG89* were detected by western blotting of worm protein extracts. However, the anti-ATXN3 also detected a band in non-transgenic wild-type N2 animals (Fig. 1C). *atx-3* encodes a deubiquitylating enzyme that is the highly conserved *C. elegans* orthologue of human *ATXN3* (Kawaguchi et al., 1994; Echtermeyer et al., 2011), thus it can be detected by western blotting with a human specific ATXN3 antibody. To confirm this, we conducted western blotting experiments using the anti-ATXN3 antibody and protein extracts from *atx-3* null mutants. *atx-3(tm1689)* is a loss-of-function mutation consisting of a 660 bp deletion along with a 6 bp insertion (C. elegans Deletion Mutant Consortium, 2012; Rodrigues et al., 2007), and no signal was observed. These data suggest that the anti-ATXN3 antibody recognizes *C. elegans* ATX-3 in N2 wild-type worms and partly obscures the signal for *ATXN3-CAG10* transgenics, but a specific, higher molecular weight signal is visible in extracts from *ATXN3-CAG89* animals (Fig. 1C). Finally, we confirmed the specificity of the anti-ATXN3 antibody by western blotting using protein extracts from cells originating from healthy controls or MJD patients (Fig. 1D).

### Decreased lifespan and impaired neuronal phenotypes in mutant *ATXN3* transgenics

**Fig. 2.**
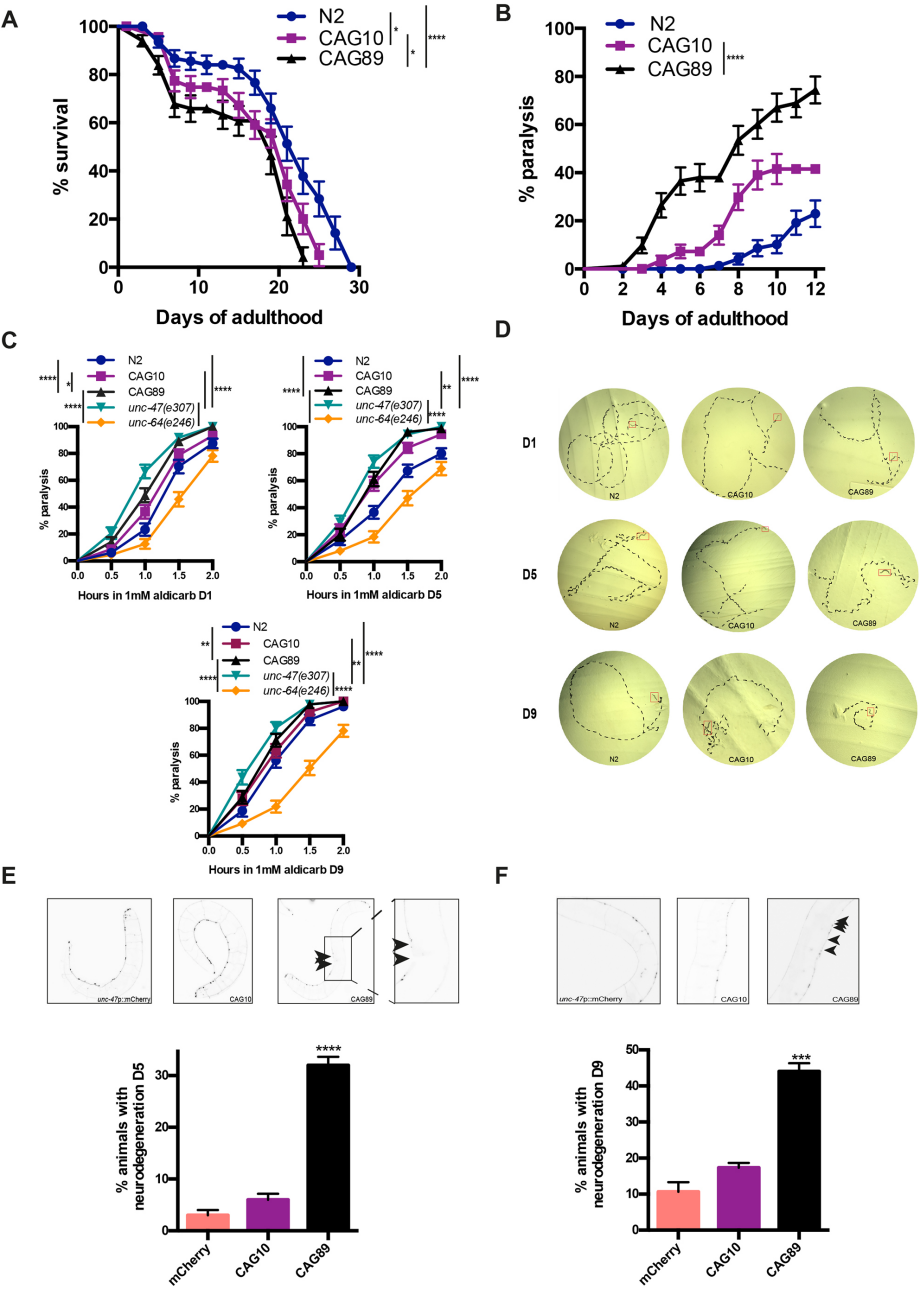
**Lifespan and neuronal phenotypes in *ATXN3* transgenics.** (A) *ATXN3-CAG89* worms had reduced lifespan compared with wild-type *ATXN3-CAG10* or N2 worms [*P<0.05 and ****P<0.0001, respectively, by log-rank (Mantel–Cox) test; *n*=300-360]. *ATXN3-CAG10* animals showed reduced lifespan compared with N2 wild-type worms [*P<0.05, by log-rank (Mantel–Cox) test; *n*=300-360]. The experiment was repeated three times. (B) CAG89 transgenics had a significantly higher paralysis phenotype compared with wild-type *ATXN3-CAG10* transgenics [****P<0.0001, by log-rank (Mantel–Cox) test; *n*=270-300]. The experiment was repeated three times. (C) Cholinergic neuronal transmission was measured by determining the onset of paralysis induced by the cholinesterase inhibitor aldicarb. Adult day 1 *unc-47(e307)* and *ATXN3-CAG89* mutant strains showed a higher hypersensitive phenotype in the presence of the aldicarb-induced paralysis compared with wild-type N2, *ATXN3-CAG10* and *unc-64(e246)* worms [****P<0.0001 for *unc-47(e307)* mutant worms when compared with the controls, and ****P<0.0001, *P<0.05 and ****P<0.0001 for *ATXN3-CAG89* mutants compared with the controls, by log-rank (Mantel–Cox) test; *n*=270-300]. Adult day 5 *unc-47(e307)* and *ATXN3-CAG89* worms also showed a higher hypersensitive phenotype in presence of the aldicarb-induced paralysis compared with wild-type N2, *ATXN3-CAG10* and *unc-64(e246)* worms [****P<0.0001, **P<0.01 and ****P<0.0001 for *unc-47(e307)* mutants, respectively, compared with the controls, and ****P<0.0001 for *ATXN3-CAG89* mutants when compared with wild-type N2 and *unc-64(e246)* worms, by log-rank (Mantel–Cox) test; *n*=270-300]. A hypersensitive phenotype was observed at adult day 9 *unc-47(e307)* and *ATXN3-CAG89* mutant strains when compared with wild-type N2, *ATXN3-CAG10* and *unc-64(e246)* worms [****P<0.0001, **P<0.01 and ****P<0.0001 for *unc-47(e307)* mutant worms when compared with the controls, respectively, and **P<0.01 and ****P<0.0001 for *ATXN3-CAG89* mutants when compared with the wild-type N2 and *unc-64(e246)* worms, by log-rank (Mantel–Cox) test; *n*=270-300]. The experiment was repeated three times. (D) Synchronized adult day 1, 5 and 9 worms were placed on NGM plates and photographed after 10 min of free movement. *ATXN3-CAG89* mutant worms showed defects in motility when compared with wild-type N2 and *ATXN3-CAG10* transgenic worms. (E,F) Representative photos of living, adult expressing wild-type *unc-47*p::mCherry, *unc-47*p::mCherry; *ATXN3-CAG10* and *unc-47*p::mCherry; *ATXN3-CAG89* transgenic worms at days 5 and 9 of adulthood. Images of the GABAergic motoneurons from entire *unc-47*p::mCherry and *unc-47*p::mCherry; *ATXN3-CAG10* worms were taken. Image of an entire *unc-47*p::mCherry; *ATXN3-CAG89* transgenic worm was taken and then zoomed in the panel on the right, representing a magnification of the area indicated. Increased incidences of gaps or breakages were observed in mutant *unc-47*p::mCherry; *ATXN3-CAG89* transgenics compared with wild-type *unc-47*p::mCherry and *unc-47*p::mCherry; *ATXN3-CAG10* controls. Quantification of neurodegeneration in transgenic worms at days 5 and 9 of adulthood. *ATXN3-CAG89* transgenics showed a significant increase of neurodegeneration compared with *unc-47*p::mCherry and *ATXN3-CAG10* controls (****P<0.0001 for day 5 of adulthood and ***P<0.001 for adult dav 9 worms, by Student's unpaired *t-*test; *n*=100 for each condition). These experiments were replicated three times.

We wondered whether the expression of non-native proteins led to decreased health in the transgenic strains. Initially, we investigated whether lifespan was altered in our *ATXN3* transgenics. Using age-synchronized animals we observed that both *ATXN3-CAG10* and *ATXN3-CAG89* transgenics showed significantly decreased lifespan compared with non-transgenic wild-type N2 worms (Fig. 2A). These data demonstrate that increased expression of *ATXN3* transgenes has negative consequences on lifespan, but the mutant *ATXN3-CAG89* transgene had a more severe phenotype compared with *ATXN3-CAG10* controls.

Decreased lifespan can indicate poor health of the animals, and one sign of ageing in *C. elegans* is decreased motility that can be quantified using assays for progressive, age-dependent paralysis (Collins et al., 2008; Herndon et al., 2002). We observed that the age-synchronized *ATXN3-CAG89* transgenics displayed progressive motor defects compared with wild-type *ATXN3-CAG10* transgenics. Starting during adulthood, *ATXN3-CAG89* transgenics displayed uncoordinated motility phenotypes, progressing to paralysation over a period of 12 days, and occurred at a higher rate compared with *ATXN3-CAG10* controls (Fig. 2B).

The nematode *C. elegans* body wall muscle cells receive excitatory (acetylcholine) and inhibitory (GABA) inputs to coordinate muscle contraction and relaxation and to facilitate movement (McIntire et al., 1993). Activity of the neuromuscular junction can be measured indirectly with the acetylcholinesterase inhibitor aldicarb (Mahoney et al., 2006). It has been shown that treating worms with the known compound, aldicarb, results in accumulation of acetylcholine at neuromuscular junctions, leading to a number of different phenotypes: hyperactive cholinergic synapses, muscle hypercontraction and acute paralysis (Mahoney et al., 2006). Mutant strains having a defect in synaptic vesicle release demonstrate resistance for aldicarb. Resistance or hypersensitivity to aldicarb-induced paralysis has been used to identify genes that regulate acetylcholine secretion or inhibitory GABA signalling in different studies, including ours for ALS models (Loria et al., 2004; Vaccaro et al., 2012b). To investigate whether our transgenic *ATXN3* strains had abnormal activity at the neuromuscular junction, we exposed them and two control strains, *unc-64(e246)* (resistant to aldicarb, encodes syntaxin) and *unc-47(e307)* (hypersensitive to aldicarb), to aldicarb. Transgenic *ATXN3-CAG89* strains and *unc-47(e307)* mutants were hypersensitive to aldicarb-induced paralysis compared with *ATXN3-CAG10* transgenics, wild-type N2 worms, and *unc-64(e246)* mutants at days 1, 5 and 9 of adulthood (Fig. 2C). *ATXN3-CAG10* transgenics did show a hypersensitivity to aldicarb-induced paralysis, but this was less severe than what was observed for *ATXN3-CAG89* animals. These data suggest that the function of the GABAergic motoneurons, and perhaps their inhibitory signalling function, is impaired in mutant *ATXN3-CAG89* transgenics.

Additionally, we tracked the movement of wild-type N2 worms and both *ATXN3* transgenics for a period of 10 min on agar plates. We observed that *ATXN3-CAG89* worms had impaired motility phenotypes and explored less of their area compared with wild-type N2, and *ATXN3-CAG10* worms at days 1, 5 and 9 of adulthood (Fig. 2D). Overall, these data demonstrate that mutant *ATXN3-CAG89* transgenic worms have increased neuromuscular dysfunction that advances in a progressive manner, leading to increased rates of paralysis compared with controls.

### Mutant *ATXN3-CAG89* causes progressive motoneuron degeneration

Many neurodegenerative diseases are characterized by neuronal dysfunction before degeneration (Saxena and Caroni, 2011). To determinate whether the progressive paralysis phenotype observed in *ATXN3-CAG89* worms was accompanied by neurodegeneration, we crossed the transgenic lines with an integrated reporter, *unc-47*p::mCherry, expressing the red fluorescent protein mCherry in GABAergic motoneurons (Petrash et al., 2013). We observed a significant increase of gaps and/or breaks in motoneurons of *ATXN3-CAG89* worms when compared with the wild-type *unc-47*p::mCherry and *ATXN3-CAG10* transgenics at days 5 and 9 of adulthood (Fig. 2E,F). We did not observe neurodegeneration in young adult day 1 mutant *ATXN3-CAG89* transgenic worms (Fig. S1B). These observations suggest a gradual decline of neuronal function that is correlated with age-dependent neurodegeneration as observed in diseases such as MJD.

We investigated whether endogenous *atx-3* contributed to motility and neurodegeneration phenotypes in our *ATXN3-CAG89* transgenics. We observed that the rates of paralysis and neurodegeneration of *atx-3(tm1689)*; *ATXN3-CAG89* animals was indistinguishable from *ATXN3-CAG89* controls, suggesting that *atx-3* does not contribute to *ATXN3-CAG89* phenotypes (Fig. S1).

As *ATXN3* and mCherry are both expressed under the same promoter (*unc-47*), we wondered whether transcription factor depletion could contribute to the motility phenotypes observed in our *ATXN3-CAG89* transgenics. To investigate, we turned to a worm tracking system (*Wmicrotracker*; Phylum Tech) able to measure both automatically and simultaneously the movement of a population of worms placed in 96-well microtitre plates over several hours (Veriepe et al., 2015; Vaccaro et al., 2012a; Therrien and Parker, 2014; Schmeisser et al., 2017). The apparatus makes use of two infrared light beams crossing each microtitre well from top to bottom, and a detector determines how often the light rays are interrupted by worms moving in the well. Each interruption counts as a movement registered by the machine (Schmeisser et al., 2017). We observed no difference in overall movement between *ATXN3-CAG89* and *unc-47*p::mCherry; *ATXN3-CAG89* worms (Fig. S2). These data suggest that the addition of the *unc-47*p::mCherry transgene to *ATXN3-CAG89* transgenics does not influence motility phenotypes.

### Methylene Blue, salubrinal and guanabenz suppress paralysis and extend lifespan in *ATXN3-CAG89* transgenics without affecting the expression of this transgene

**Fig. 3.**
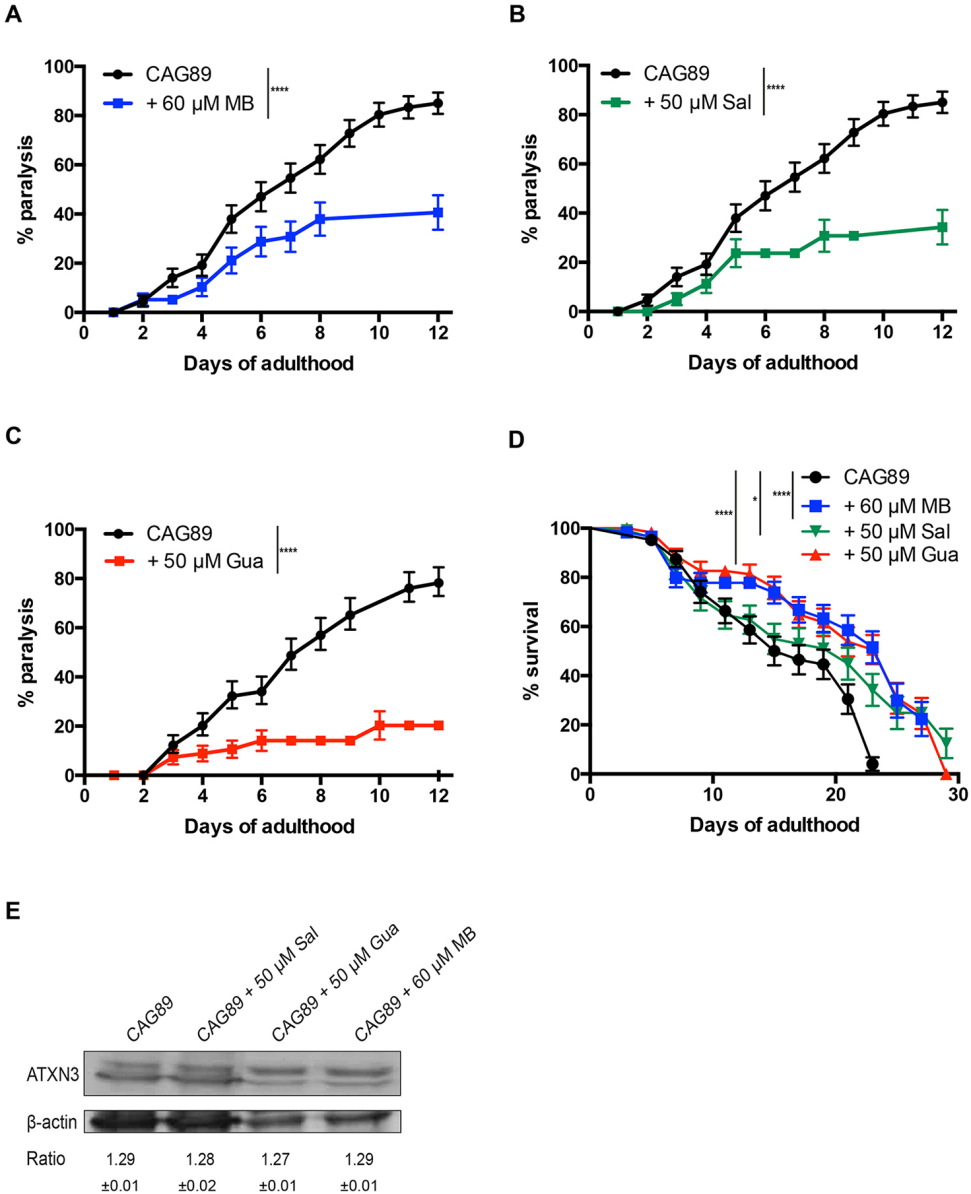
**Methylene Blue, salubrinal or guanabenz** **suppresses** **paralysis during** **ageing and extends** **lifespan in *ATXN3-CAG89* worms without affecting the expression level of this transgene.** (A-C) The motor defect phenotype observed in *ATXN3-CAG89* worms was significantly decreased in the presence of 60 μM MB (****P<0.0001), 50 µM Sal (****P<0.0001) or 50 µM Gua (****P<0.0001) [by log-rank (Mantel–Cox) test; *n*=270-300]. This experiment was replicated three times. (D) *ATXN3-CAG89* worms showed an increase lifespan in the presence of 60 μM MB (****P<0.0001), 50 µM Sal (*P<0.05) or 50 µM Gua (****P<0.0001) [by log-rank (Mantel–Cox) test; *n*=300-360]. This experiment was replicated three times. (E) Total protein levels for transgenic worms expressing mutant *ATXN3-CAG89* with and without exposure to Sal, Gua or MB. Antibody detection revealed signals for all four conditions. No differences in expression were observed between the untreated and treated transgenic worms. Actin staining was used as a loading control, and the expression ratio±s.e.m. of ATXN3 to actin was determined from three independent experiments. Representative western blots are shown. Gua, guanabenz; MB, Methylene Blue; Sal, salubrinal.

Our group previously identified several small molecules, including Methylene Blue, salubrinal and guanabenz, that target the ER stress response and were shown to have beneficial effects against mutant TDP-43 neuronal toxicity in models for ALS (Vaccaro et al., 2012a, 2013) and to have protective effects in models for hereditary spastic paraplegia (Julien et al., 2016). Thus, we wondered whether these compounds had neuroprotective activity against mutant *ATXN3-CAG89*. We tested these compounds and found that all three molecules suppressed the age-dependent paralysis phenotype caused by mutant *ATXN3-CAG89* (Fig. 3A-C), and extended the lifespan of these transgenic worms (Fig. 3D). Additionally, we wondered whether these compounds had any effect on the expression of *ATXN3-CAG89* transgenes, perhaps influencing paralysis and lifespan phenotypes. We confirmed that these compounds did not affect the expression of *ATXN3-CAG89* transgenes by western blotting with a human specific anti-ATXN3 antibody (Fig. 3E).

**Fig. 4.**
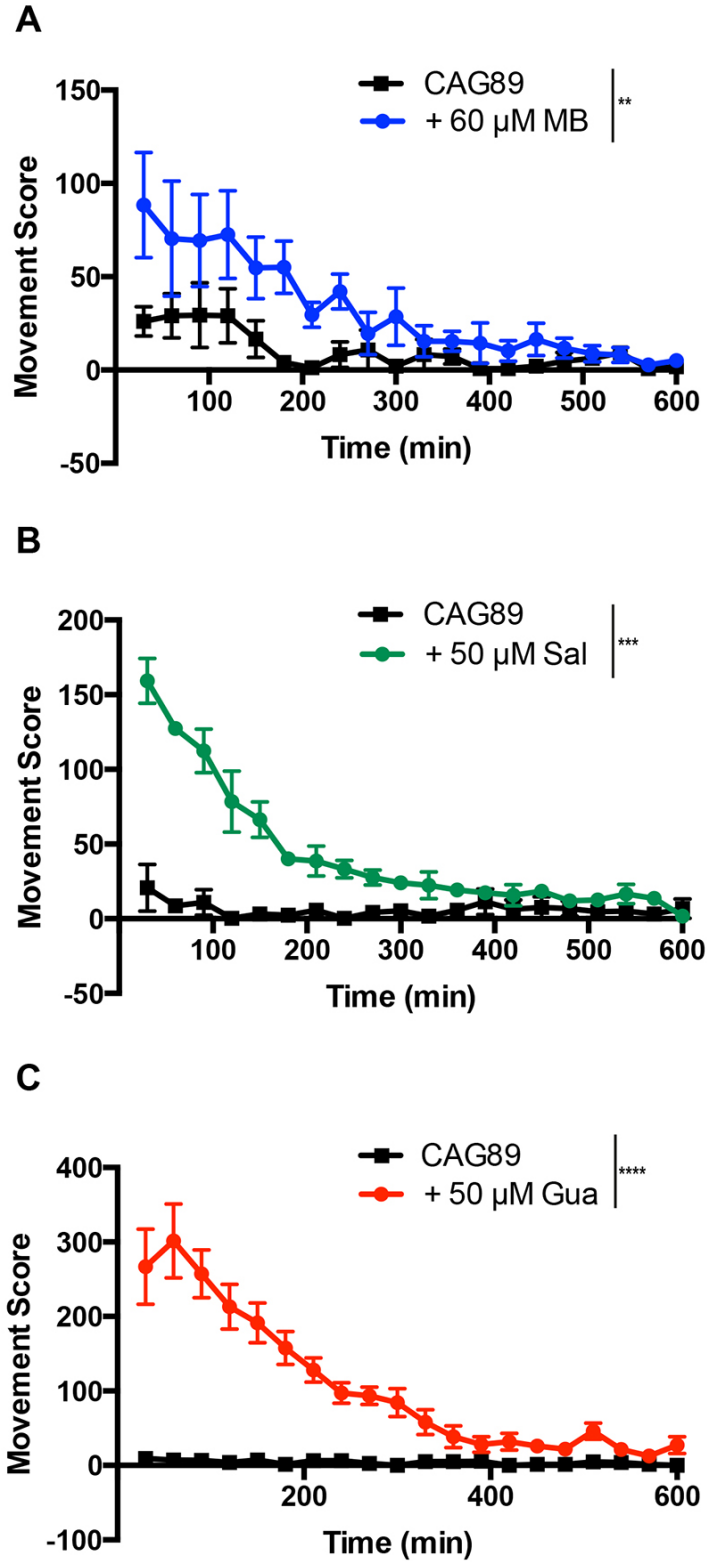
**Methylene Blue, salubrinal or guanabenz** **suppresses** **acute paralysis in *ATXN3-CAG89* transgenics.** The swimming activity of *ATXN3-CAG89* worms was scored for a period of 10 h, and treatment with (A) 60 μM MB (**P<0.01), (B) 50 µM Sal (***P<0.001) or (C) 50 µM Gua (****P<0.0001) (by Student's unpaired *t-*test) significantly rescued the impaired movement phenotype of *ATXN3-CAG89* worms. This experiment was done three times. Gua, guanabenz; MB, Methylene Blue; Sal, salubrinal.

To confirm the neuroprotective activity of these molecules with a separate approach, we turned to our automated assay measuring the movement of *C. elegans* grown in liquid culture (Veriepe et al., 2015; Vaccaro et al., 2012a; Therrien and Parker, 2014). We observed that *ATXN3-CAG89* animals treated with Methylene Blue, salubrinal or guanabenz had increased motility compared with untreated controls (Fig. 4). These data suggest that molecules regulating the ER stress response can attenuate neuronal dysfunction caused by mutant *ATXN3-CAG89*.

### Small molecules rescue neurodegeneration in *ATXN3-CAG89* transgenics

**Fig. 5.**
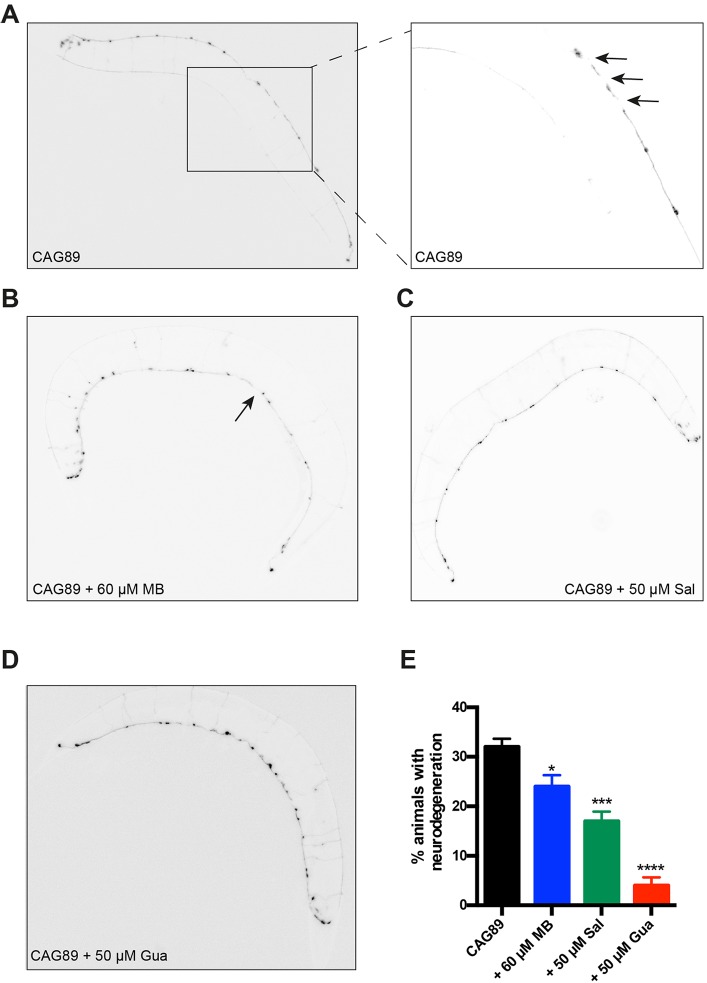
**Rescue of** **motoneuron** **degeneration by small molecules in *ATXN3-CAG89* transgenics.** Shown are representative photos of living, adult *unc-47*p::mCherry; *ATXN3-CAG89* transgenics at day 5 of adulthood with or without compounds. Images are black and white, photo-reversed to aid visualization of neurons. (A) Image of degenerating GABAergic motoneurons from an entire *ATXN3-CAG89* transgenic. The panel on the right is a magnification of the area indicated by the rectangle. Arrows indicate gaps or breaks along neuronal processes. Rescue of neurodegeneration was observed in *ATXN3-CAG89* worms in the presence of (B) 60 μM MB, (C) 50 µM Sal or (D) 50 µM Gua. (E) Quantification of neurodegeneration in transgenic *ATXN3-CAG89* worms at day 5 of adulthood. Significant rescue of the neurodegeneration morphology was observed in *ATXN3-CAG89* transgenics when treated with 60 μM MB (*P<0.05), 50 µM Sal (***P<0.001) or 50 µM Gua (****P<0.0001) (by Student's unpaired *t-*test, *n*=100 for each condition). These experiments were repeated three times. Gua, guanabenz; MB, Methylene Blue; Sal, salubrinal.

After observing that Methylene Blue, salubrinal or guanabenz suppressed mutant *ATXN3-CAG89*-induced paralysis, we examined whether these compounds had protective effects against motoneuron degeneration. Using the *unc-47*p::mCherry; *ATXN3-CAG89* strain, we visualized the GABAergic motoneurons *in vivo* in day 5 adults and observed a significant decrease of neurodegeneration for worms when treated with any of the three compounds (Fig. 5). These data demonstrate that chemical manipulation of ER stress mechanisms protects neurons against mutant ATXN3 toxicity.

### Methylene Blue, salubrinal and guanabenz prevent the oxidative stress induced by *ATXN3-CAG89* transgenics

**Fig. 6.**
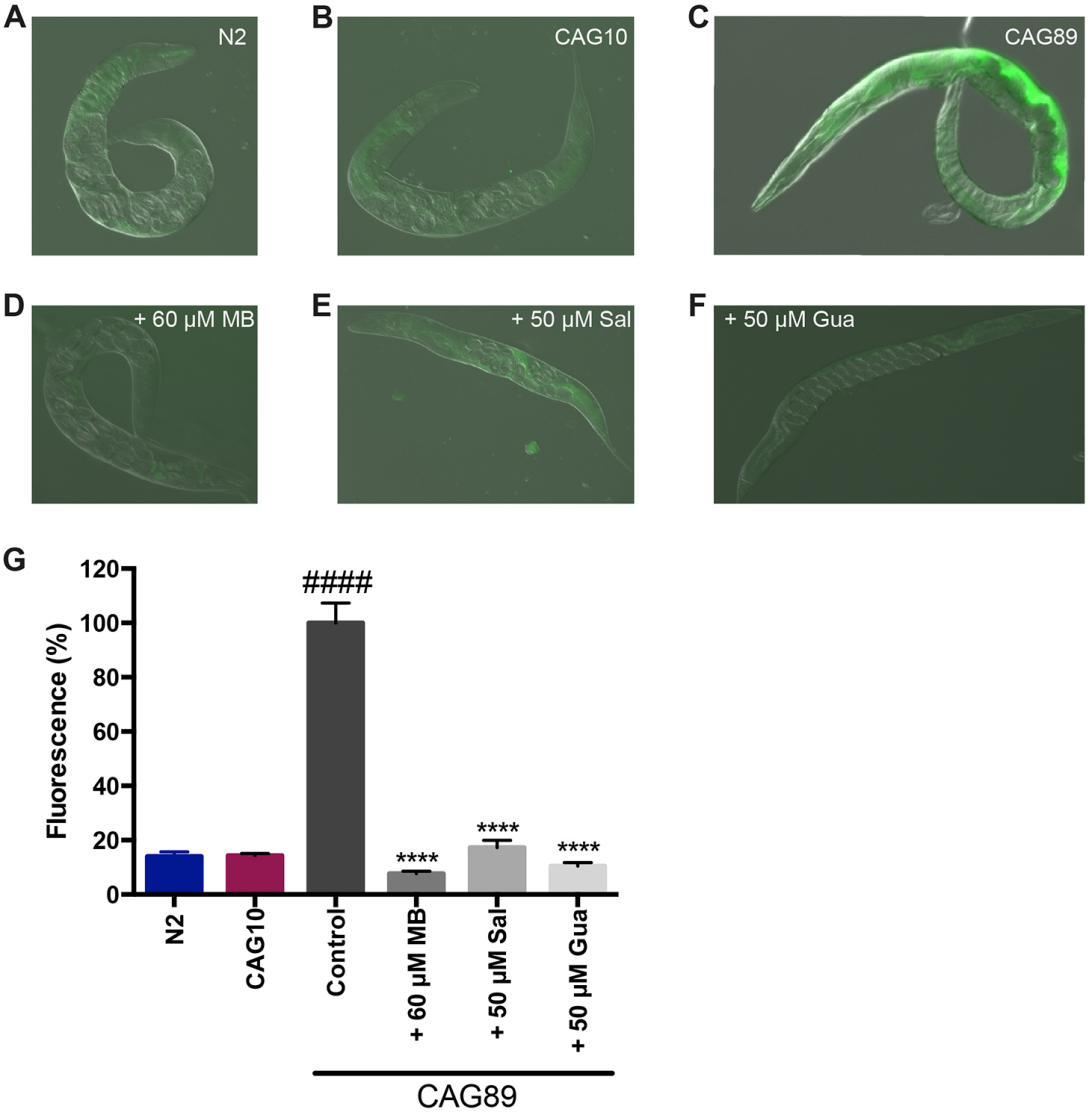
**Reduction of oxidative stress in *ATXN3-CAG89* transgenics by** **Methylene Blue, guanabenz or salubrinal.** Shown are representative photos of living, adult (A) wild-type N2 worms and (B) wild-type *ATXN3-CAG10* and (C) mutant *ATXN3-CAG89* transgenics at day 2 of adulthood in the presence of the oxidative stress marker, 2′,7′-dichlorofluorescein diacetate (DCF-DA). Increased fluorescence is observed in the (C) *ATXN3-CAG89* transgenic worms. (D-F) *ATXN3-CAG89* transgenics stained with DCF-DA showed decreased fluorescence after treatment with (D) 60 μM MB, (E) 50 µM Sal or (F) 50 µM Gua. (G) Quantification of fluorescence in N2 controls, *ATXN3-CAG10* or *ATXN3-CAG89* transgenics stained with DCF-DA. *ATXN3-CAG89* transgenics showed increased fluorescence compared with N2 or *ATXN3-CAG10* worms (^####^*P*<0.0001). *ATXN3-CAG89* transgenics had less fluorescence when treated with 60 μM MB, 50 µM Sal or 50 µM Gua in the presence of DCF-DA compared with untreated CAG89 controls (****P<0.0001) (Student's unpaired *t*-tests, *n*=17-25 for each condition). This experiment was repeated three times. Gua, guanabenz; MB, Methylene Blue; Sal, salubrinal.

We wondered whether one mechanism associated with mutant *ATXN3-CAG89* toxicity involved elevated levels of oxidative stress, as we have previously observed in other models of neuronal proteotoxicity (Vaccaro et al., 2013, 2012a). Global oxidative stress can be detected by staining worms with the fluorescent dye 2′,7′-dichlorofluorescein diacetate (DCF-DA), and we observed increased fluorescence in *ATXN3-CAG89* transgenics compared with wild-type N2 controls and *ATXN3-CAG10* transgenics (Fig. 6A-C).

Our previous work suggests that oxidative stress might be linked with activation of the unfolded protein response in the ER (Julien et al., 2016; Vaccaro et al., 2013). Furthermore, small molecule-mediated reduction of the ER stress response was likewise correlated with decreased oxidative stress levels in *C. elegans* proteotoxicity models. We observed that treatment of *ATXN3-CAG89* transgenics with Methylene Blue, guanabenz or salubrinal reduced the fluorescence from DCF-DA staining (Fig. 6D-G). These data suggest that the ER stress response might be involved in neuronal toxicity caused by mutant *ATXN3-CAG89*.

### Rescue of ER stress response by Methylene Blue, salubrinal and guanabenz in *ATXN3-CAG89* transgenic worms

**Fig. 7.**
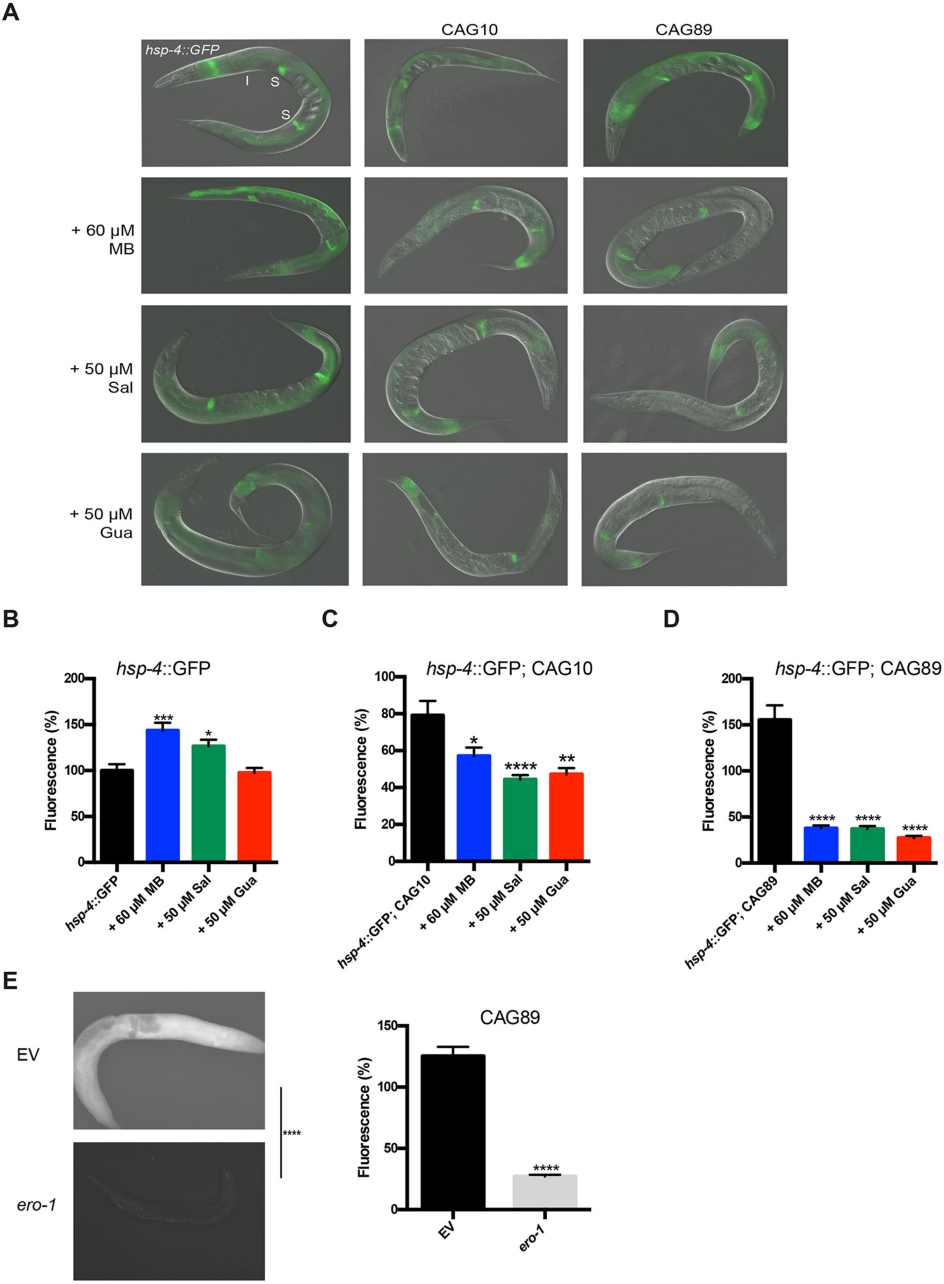
**Methylene** **Blue, guanabenz and salubrinal rescue the ER stress response in *ATXN3-CAG89* transgenic worms.** (A) Shown are representative photos of living, adult *hsp-4*::GFP, *hsp-*4::GFP; *ATXN3-CAG10* and *hsp-*4::GFP; *ATXN3-CAG89* transgenics at day 1 of adulthood. The *hsp-*4::GFP; *ATXN3-CAG89* transgenics show increased GFP expression compared with *hsp-4*::GFP, or *hsp-*4::GFP; *ATXN3-CAG10* controls (top panels). Treatment with 60 μM MB, 50 µM Sal or 50 µM Gua reduces the fluorescence of *hsp-*4::GFP; *ATXN3-CAG89* to control levels. The *hsp-4*::GFP reporter shows increased fluorescence in the intestine (I) and spermatecha (S) of adult animals. (B-D) Quantification of fluorescence of transgenics with or without treatment with compounds. Increased fluorescence was observed in *hsp*-4::GFP worms when treated with 60 μM MB (***P<0.001) or 50 µM Sal (*P<0.05) (Student’s unpaired *t*-tests, *n*=17-25 for each condition). Fluorescence was decreased in *hsp*-4::GFP; *ATXN3-CAG10* transgenic worms when treated with 60 μM MB (*P<0.05), 50 µM Sal (****P<0.0001) or 50 µM Gua (**P<0.01) (Student’s unpaired *t*-tests, *n*=17-25 for each condition). However, the decreased fluorescence observed was even more significant in *hsp*-4::GFP; *ATXN3-CAG89* mutants when treated with 60 μM MB, 50 µM Sal or 50 µM Gua (****P<0.0001) (Student’s unpaired *t*-tests, *n*=17-25 for each condition). These experiments were replicated three times. (E) Shown are representative photos of living, adult day 2 *ATXN3-CAG89* transgenics fed with *E. coli* containing an empty vector (EV) or expressing dsRNA against *ero-1*. *ATXN3-CAG89* mutant worms showed a high level of fluorescence when stained with 2′,7′-dichlorofluorescein diacetate (DCF-DA) and a significant decrease of this fluorescence when grown in the presence of *ero-1* RNAi. Quantification of fluorescence of *ATXN3-CAG89* mutant worms on EV or *ero-1* RNAi. A significant decrease of fluorescence of *ATXN3-CAG89* mutants was observed in the presence of *ero-1* RNAi when compared with EV (****P<0.0001) (Student's unpaired *t*-tests, *n*=17-25 for each condition). This experiment was repeated three times. Gua, guanabenz; MB, Methylene Blue; Sal, salubrinal.

After observing high levels of oxidative stress in *ATXN3-CAG89* transgenics, we wanted to test directly for the involvement of the ER stress response. *hsp-4* encodes a widely expressed, protective Hsp70/BiP protein induced by ER stress that can be monitored with a transgenic, transcriptional *hsp-4*::GFP reporter that shows strong fluorescence in the intestine and spermatheca (Urano et al., 2002). We crossed the *ATXN3* transgenics with the *hsp-4*::GFP reporter and observed increased fluorescence in *ATXN3-CAG89*; *hsp-4*::GFP animals compared with *ATXN3-CAG10*; *hsp-4*::GFP or *hsp-4*::GFP controls (Fig. 7A, top panels). We recently showed that Methylene Blue, guanabenz and salubrinal rescue the ER stress response in multiple models for another neurological disorder, hereditary spastic paraplegias (Julien et al., 2016). Based on this finding, we examined whether any of these compounds were able to prevent the ER stress response caused by *ATXN3-CAG89* transgenics. We found that all three compounds reduced fluorescence of the ER stress *hsp-4*::GFP reporter transgene in *ATXN3-CAG89* transgenics (Fig. 7A-D). A significant reduction of the ER stress was also observed in the *ATXN3-CAG10* transgenic worms when treated with the compounds. This is consistent with the fact that *ATXN3-CAG10* transgenics have motility and lifespan phenotypes intermediate to N2 worms and *ATXN3-CAG89* transgenics. We observed a significant induction of the GFP signal in *hsp-4*::GFP worms when treated with Methylene Blue and salubrinal, suggesting that these molecules might act by stimulating an early ER stress response outcome that might be neuroprotective in the context of *ATXN3* transgenics, ultimately resulting in lower proteotoxicity followed by decreased ER stress overall. These data suggest that small molecule interventions centred on the ER stress response protect against mutant ATXN3 toxicity.

If stress within the ER persists, and it is not resolved by the early protective ER unfolded protein response (UPR^ER^) pathways, this can stimulate the clearance of misfolded proteins from the ER through ER-associated degradation (ERAD). The processing of misfolded proteins by ERAD is a redox-intensive process that requires the ER oxidoreductase *ero-1*/ERO1, the activity of which can result in the production of peroxides and, consequently, increased reactive oxygen species (Harding et al., 2003). Thus, impairing *ero-1*/ERO1 activity disrupts the processing of proteins, along with the generation of peroxides and associated oxidative stress (Harding et al., 2003). Therefore, we predicted that knockdown of *ero-1* by RNAi could reduce the amount of oxidative stress observed in our *ATXN3-CAG89* transgenics worms. We observed that *ATXN3-CAG89* mutant worms stained with DCF-DA in the presence of *ero-1*(RNAi) showed a significant decrease of fluorescence compared with the empty vector RNAi controls (Fig. 7E). These data suggest that the oxidative stress observed in our *ATXN3-CAG89* transgenic mutant worms might originate in the ER and is dependent on the activity of *ero*-*1*.

### Chemical-genetic approach analysis for the UPR^ER^ pathways

ER stress leads to activation of the UPR by three main signalling branches, resulting in an upregulation of chaperone proteins and a general arrest of protein translation (Walker and Atkin, 2011). Based on our data demonstrating an activation of ER stress in *ATXN3-CAG89* mutants, along with a reduction of this stress by UPR^ER^-associated compounds (Fig. 7), we wondered whether these compounds required any specific UPR^ER^ branches for their neuroprotective activities. To identify the mechanisms related to each compound, we opted for a genetic approach using mutants against key components of two branches of the UPR^ER^ pathways, such as *atf-6/*ATF6, *pek-1*/PERK, and RNAi for *ire-1*/IRE1.

**Fig. 8.**
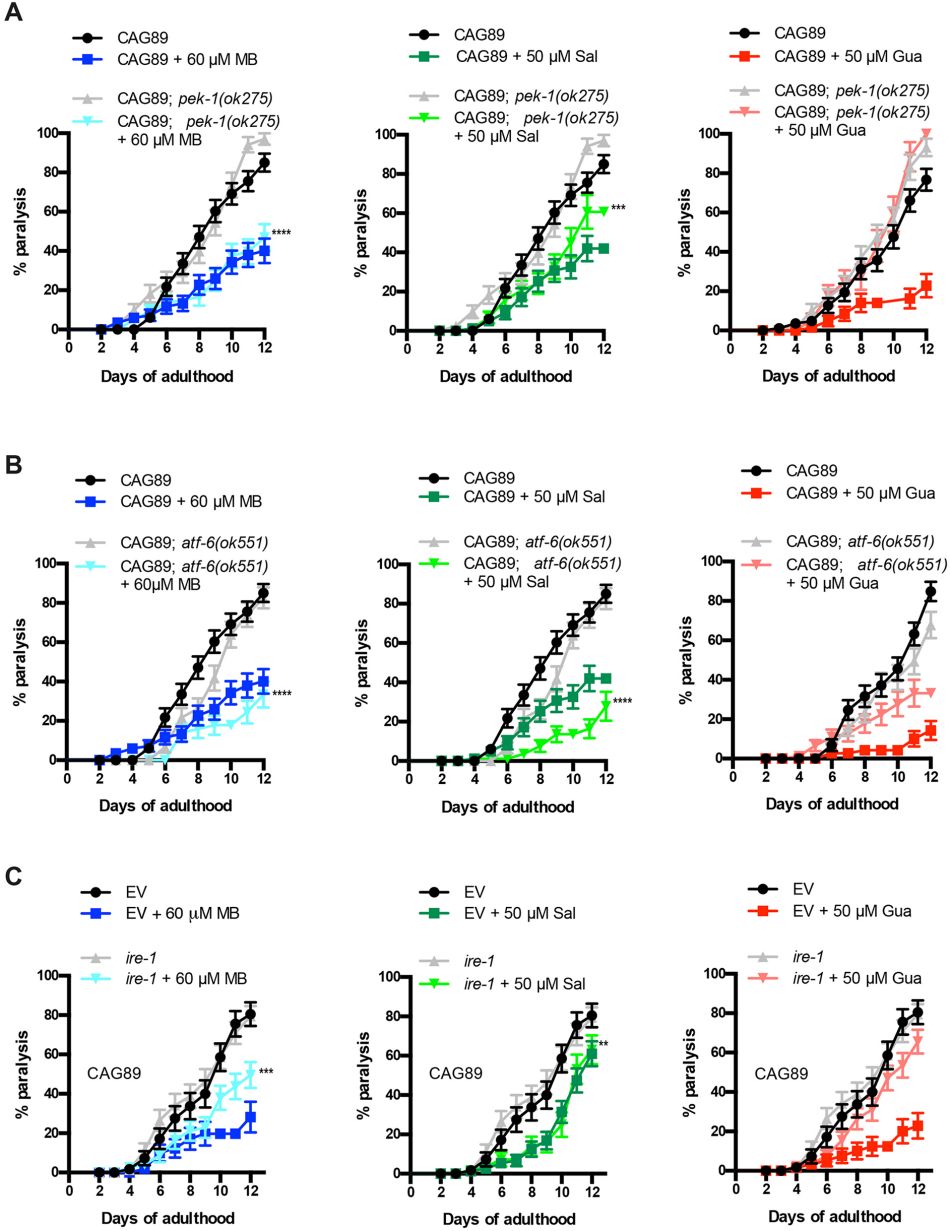
**Chemical-genetic approach analysis of UPR^ER^ pathways in *ATXN3-CAG89* mutants.** (A) Paralysis assays for *ATXN3-CAG89* and *ATXN3-CAG89*; *pek-1(ok275)* transgenic mutant worms in presence of 60 μM MB, 50 μM Sal and 50 μM Gua. We observed that Gua failed to suppress the paralysis in *ATXN3-CAG89*; *pek-1(ok275)* worms, Sal partly suppressed paralysis (***P<0.001) and MB suppressed paralysis in *ATXN3-CAG89*; *pek-1(ok275)* mutant worms (****P<0.0001) [by log-rank (Mantel–Cox) test; *n*=270-300]. This experiment was repeated three times. (B) *ATXN3-CAG89* and *ATXN3-CAG89*; *atf-6(ok551)* mutant worms were treated with 60 μM MB, 50 μM Sal and 50 μM Gua. We observed that Sal and MB suppressed paralysis in *ATXN3-CAG89*; *atf-6(ok551)* mutant worms (****P<0.0001 for both compounds), contrary to Gua, which is dependent on the *atf-6* branch of the UPR^ER^ pathway, showing an incapacity to suppress the paralysis in the transgenic mutant worms [by log-rank (Mantel–Cox) test; *n*=270-300]. This experiment was repeated three times. (C) Paralysis assays for *ire-1* RNAi *ATXN3-CAG89* worms treated with 60 μM MB, 50 μM Sal and 50 μM Gua. We observed that Gua totally and MB partly (***P<0.001) failed to suppress the paralysis in *ire-1* RNAi worms, contrary to Sal, which suppressed paralysis in *ire-1* RNAi worms (**P<0.01) [by log-rank (Mantel–Cox) test; *n*=270-300]. This experiment was repeated three times. Gua, guanabenz; MB, Methylene Blue; Sal, salubrinal; UPR^ER^, ER unfolded protein response.

We investigated the contribution of the *pek-1*/PERK pathway with the deletion mutant *pek-1(ok275).* We observed that the rescuing activity of guanabenz was completely dependent on *pek-1.* Methylene Blue continued to suppress paralysis in the absence of *pek-1*, suggesting that this compound does not require the *pek-1*/PERK branch of the UPR^ER^. Concerning salubrinal, this compound was partly dependent on *pek-1*, because we observed a reduced ability to suppress paralysis in *ATXN3-CAG89*; *pek-1(ok275)* worms (Fig. 8A).

We examined the neuroprotective activity of *atf-6* using *atf-6(ok551)* mutant worms. We observed that *atf-6* was not required for the neuroprotective activity of Methylene Blue and salubrinal, because these compounds maintained their ability to suppress paralysis in *ATXN3-CAG89*; *atf-6(ok551)* mutants. However, we observed that guanabenz failed to suppress the paralysis in *ATXN3-CAG89* mutants when crossed with *atf-6* loss-of-function mutants, confirming dependence on the *atf-6* branch of the UPR^ER^ (Fig. 8B).

To investigate the role of *ire-1* in neuroprotection, we used RNAi against *ire-1(zc14).* We observed that the rescuing activity of guanabenz was completely dependent on *ire-1*, and Methylene Blue showed a partial dependence on this branch of the UPR^ER^. However, salubrinal showed no dependence on this pathway, as this compound continued to suppress the paralysis in *ire-1*(RNAi) worms (Fig. 8C). These data suggest that the three chemical compounds use distinct, or possibly overlapping, branches of the UPR^ER^ to achieve neuroprotection against CAG-mediated proteotoxicity.

### Small molecule suppression of *ATXN3-CAG89* aggregation

**Fig. 9.**
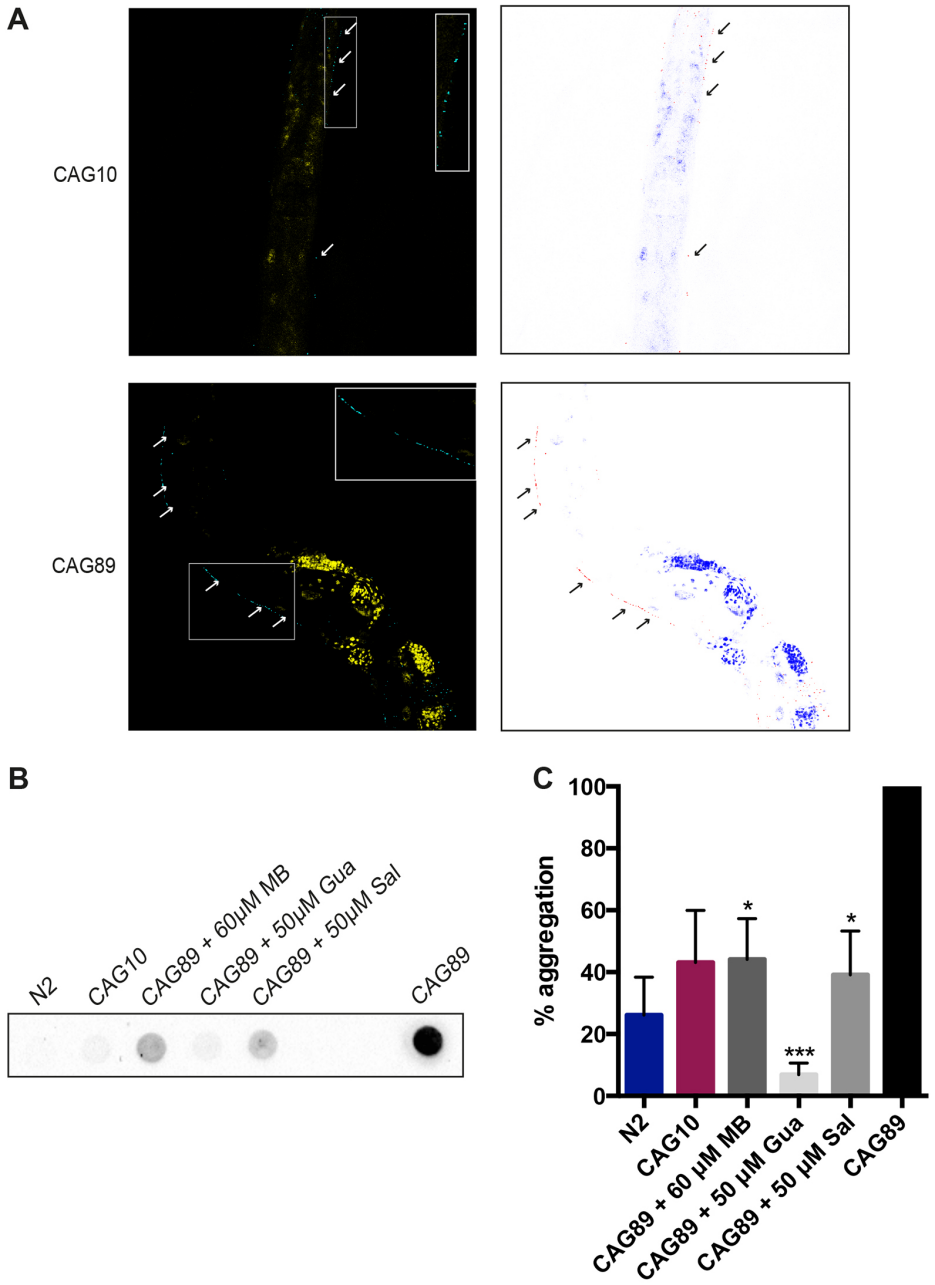
**Small molecule suppression of *ATXN3-CAG89* aggregation.** (A) Fixed whole *ATXN3-CAG10* and *ATXN3-CAG89* transgenic worms stained with a human ATXN3 antibody (blue) and TOPRO dye (yellow) for the nucleic acids. Arrows indicate ATXN3 protein in the motoneurons of *ATXN3* transgenics. Second panel is a colour-inverted image to aid visualization. The insets are magnifications of the aggregates indicated in the transgenic worms with a dashed line box. (B) Total protein levels for N2 and transgenic worms expressing *ATXN3-CAG10* or mutant *ATXN3-CAG89*. Antibody detection revealed high accumulation of the *ATXN3-CAG89* in the pelleted, insoluble fraction, when compared with *ATXN3-CAG10* and N2 wild-type worms. There was a reduction of aggregation in *ATXN3-CAG89* worms when treated with 60 μM MB, 50 μM Sal or 50 μM Gua. Gua was especially potent in reducing protein aggregation. (C) Quantification of the intensity of the aggregation in transgenic *ATXN3-CAG89* mutants and wild-type N2 and *ATXN3-CAG10* worms. There was a significant increase of aggregation in *ATXN3-CAG89* when compared with the wild-type N2 and *ATXN3-CAG10* worms. After treatment with 60 μM MB (*P<0.05), 50 µM Sal (*P<0.05) or 50 µM Gua (***P<0.001) (by ANOVA) *ATXN3-CAG89* showed a significant decrease in aggregation levels. Gua, guanabenz; MB, Methylene Blue; Sal, salubrinal.

As ATXN3 has been observed to form protein aggregates in several models, including *C. elegans*, we wondered whether our transgenics displayed similar aggregation phenotypes (Teixeira-Castro et al., 2015, 2011; Koch et al., 2011; Breuer et al., 2010). To determine whether ATXN3 proteins could be detected *in vivo*, we fixed whole *ATXN3-CAG10* and *ATXN3-CAG89* transgenics and stained them with a human ATXN3 antibody. We detected ATXN3 protein in the motoneurons of *ATXN3* transgenics (Fig. 9A). Our cursory visual examination suggested a more intense puncta signal in *ATXN3-CAG89* transgenics that might reflect increased aggregation potential of ATXN3-CAG89 protein compared with ATXN3-CAG10.

We investigated further using a biochemical assay to detect protein aggregation. Homogenized protein extracts from transgenic worms were separated into supernatant (detergent soluble) and pellet (detergent insoluble). Dot blotting the *ATXN3-CAG89* transgenic mutants with human ATXN3 antibody showed a high accumulation of the *ATXN3-CAG89* in the pelleted, insoluble fraction, when compared with *ATXN3-CAG10* and N2 wild-type worms (Fig. 9B). We then investigated whether Methylene Blue, salubrinal and guanabenz were able to reduce the aggregation observed in our *ATXN3-CAG89* transgenics. We observed a significant decrease of aggregation in *ATXN3-CAG89* worms when treated with these three compounds, and noted that guanabenz was especially potent in reducing protein aggregation (Fig. 9B,C). These data suggest neuroprotective roles for Methylene Blue, salubrinal and guanabenz in reducing mutant ATXN3 aggregation.

## DISCUSSION

We introduce new *C. elegans* models for investigating mechanisms of motoneuron toxicity caused by a polyglutamine expansion in ATXN3. To model human disease, we expressed full-length human *ATXN3* without additional tags, because the inclusion of tags can mask or enhance the phenotypes of wild-type and mutant proteins (Catoire et al., 2008; Wang et al., 2009). We engineered strains expressing *ATXN3* in the animal's 26 GABAergic neurons, because impaired activity of these motoneurons can lead to easily identifiable motility phenotypes useful for screening purposes (McIntire et al., 1997, 1993). Additionally, MJD disease is in part associated with dysfunction of motoneurons (França et al., 2008; Kanai and Kuwabara, 2009; Pinto and De Carvalho, 2008; Rub et al., 2002). Furthermore, we have previously constructed neurodegeneration models based on the expression of human disease proteins in these same GABAergic motoneurons and found that the transgenic models provide robust phenotypes for experimentation, as we have previously demonstrated in our ALS models (Vaccaro et al., 2012a). Lastly, *C. elegans* has an ATXN3 orthologue, ATX-3, that shares many cellular functions (Kawaguchi et al., 1994; Rodrigues et al., 2007; Piano et al., 2002), which perhaps extend to the nervous system, making the worm an appropriate model for studying conserved pathophysiological mechanisms of MJD.

*ATXN3-CAG89* transgenic worms showed a progressive age-dependent paralysis, and reduced lifespan phenotypes compared with wild-type *ATXN3-CAG10* transgenics. The fact that we observed a decrease of lifespan resulting from the expression of *ATXN3* transgenes in GABAergic motoneurons suggests that there might be some sort of communication from the nervous system to the rest of the organism that negatively modifies health. We and others have observed that cellular stress originating in specific neuronal populations can affect organism-wide stress mechanisms that in turn can affect lifespan (Veriepe et al., 2015; Vaccaro et al., 2012c; Taylor and Dillin, 2013; van Oosten-Hawle et al., 2013). Future studies will help to elucidate how the neuronal expression of ATXN3 regulates lifespan.

Consistent with the reduced motility observed in *ATXN3-CAG89* transgenics, we also observed significant neurodegeneration in *ATXN3-CAG89* transgenics compared with *ATXN3-CAG10* controls. These data demonstrate that the *ATXN3-CAG89* transgenics produce phenotypes distinct from wild-type *ATXN3-CAG10* and that these mutant animals might be suitable for modifier screening. Our western blotting experiments (Fig. 1C) raise the possibility that the expression of *ATXN3-CAG89* could decrease the expression of the endogenous *C. elegans atx-3* gene, perhaps accounting for some of the negative phenotypes observed in *ATXN3-CAG89* transgenics. We think this is unlikely because the western blotting data show that the level of endogenous ATX-3 in non-transgenic N2 worms (ratio 1.43) is lower than that of *ATXN3-CAG10* controls (ratio 1.84), yet N2 worms do not have motility defects or paralysis phenotypes at a rate higher than the *ATXN3-CAG10* transgenics. Furthermore, as we suspect that the anti-ATXN3 antibody detects ATX-3 and ATXN3, it is difficult to conclude definitively that the levels of endogenous ATX-3 are meaningfully different in the respective strains. Future investigations with more specific antibodies might help to resolve this issue.

Several transgenic *C. elegans* *ATXN3* models have been reported, consisting of full-length or fragments of ATXN3, a variety of CAG repeat lengths, with the protein often fused to a fluorescent marker, and high copy expression in body wall muscle cells or the entire nervous system (Khan et al., 2006; Bonanomi et al., 2014; Teixeira-Castro et al., 2011; Christie et al., 2014). The novelty of our approach is that we have expressed full-length, untagged human *ATXN3* at low levels based on MoSCi transposon-mediated single copy insertion where transgenics express only two copies of the *ATXN3* transgene. Furthermore, these transgenics have been engineered to express wild-type or mutant ATXN3 in a subset of the worm's motoneurons, resulting in strong phenotypes from a small number of cells. We believe this approach will aid drug screening, where any particular compound might need to be active in only a small number of neurons to allow for detection of suppression. Furthermore, motoneuron degeneration has been associated with MJD (França et al., 2008; McIntire et al., 1993, 1997; Pinto and De Carvalho, 2008; Kanai and Kuwabara, 2009; Rub et al., 2002).

To explore the potential of our *ATXN3* transgenics in chemical modifier screens, we tested whether we could suppress mutant *ATXN3-CAG89* phenotypes with known neuroprotective compounds. Considering the evidence linking *ATXN3* containing expanded CAG repeats to ER stress (Rodrigues et al., 2011; Evers et al., 2014; Costa and Paulson, 2012; Matos et al., 2011; Reina et al., 2010), and our previous findings connecting ER stress to other neurodegenerative diseases (Vaccaro et al., 2012a, 2013; Julien et al., 2016), we investigated whether pharmacological interventions centred on ER stress could protect against mutant *ATXN3-CAG89* toxicity. We focused on three compounds, guanabenz, salubrinal and Methylene Blue, for their known neuroprotective activity and their roles as ER stress modulators (Matos et al., 2011; Rodrigues et al., 2011; Vaccaro et al., 2013, 2012a). Encouragingly, we observed that the three compounds rescued motility defects, reduced lifespan, neurodegeneration and aggregation in animals expressing mutant *ATXN3-CAG89*. Moreover, these three compounds were also able to prevent the oxidative stress and the ER stress response induced in *ATXN3-CAG89* transgenics. Of the three compounds tested, we observed that guanabenz was the most effective at suppressing motility defects, neurodegeneration and protein aggregation phenotypes in *ATXN3-CAG89* animals. Finally, we identified the branches of the UPR^ER^ pathways essential for neuroprotection against mutant ATXN3. It is worth noting that mutations in individual UPR^ER^ genes (*ire-1*, *pek-1* or *atf-6*) did not exacerbate *ATXN3-CAG89* toxicity, suggesting that there might be redundancy between the pathways. This is consistent with the notion that the compounds tested activate the UPR^ER^ pathway to reduce *ATXN3-CAG89* toxicity. Thus, disabling individual genes blocks the activity of some molecules and not others, and helps to define the genetic requirements for neuroprotection by small molecules. However, we do predict that a triple mutant strain (*ire-1*, *pek-1* and *atf-6*) would enhance *ATXN3-CAG89* toxicity and block the rescuing activity of all molecules tested here.

Additional studies are required to extend these findings to mammalian models of MJD. However, from a practical perspective, given that guanabenz has US Food and Drug Administration approval and is being tested in a clinical trial for multiple sclerosis, it could be translated rapidly into clinical settings for MJD.

Future studies will make use of unbiased drug screen approaches to identify additional neuroprotective molecules. The novelty of this approach relies on rapidly identifying molecules that restore movement in *ATXN3-CAG89* transgenics, followed by systematic characterization of lifespan, neurodegeneration and aggregation phenotypes. Our *C. elegans* ATXN3 strains might serve as the initial step of an *in vivo* drug discovery and development pipeline for MJD and other polyglutamine diseases.

## MATERIALS AND METHODS

### Nematode strains

Standard methods of culturing and handling worms were used (Stiernagle, 2006). Worms were maintained on standard nematode growth media (NGM) plates streaked with OP50 *Escherichia* *coli*. All strains were scored at 20°C. Strains used for this study were as follows: N2, *atx-3(tm1689)*, *unc-47(e307)*, *unc-64(e246)*, *zcls4*[*hsp-4*::GFP], *atf-6(ok551)* and *pek-1(ok275)*; all obtained from the *C. elegans* Genetics Center (University of Minnesota, Minneapolis).

### Transgenic *ATXN3* worms and plasmid constructs

Human cDNAs for wild-type and mutant *ATXN3* were obtained from Dr Guy Rouleau (Montreal Neurological Institute and Hospital, McGill University). The cDNAs were amplified by PCR and cloned into the Gateway vector pDONR221 following the manufacturer's protocol (Invitrogen). Multisite Gateway recombination was performed with the pDONR *ATXN3* clones along with clones containing the *unc-47* promoter (obtained from Dr Erik Jorgensen, University of Utah), the *unc-*54 3′UTR plasmid pCM5.37 (obtained from Dr Geraldine Seydoux, Johns Hopkins; Addgene plasmid 17253) and the destination vector pCFJ150 to create *unc-47*p::mCherry; *ATXN3-CAG10* and *unc-47*p::mCherry; *ATXN3-CAG89* expression vectors. Transgenic lines were created by microinjection of *unc-119(ed3)* worms and screened for MoSCi transposon-mediated single copy insertion of the desired transgene. Several lines were isolated for each transgene of interest, and those showing similar behaviour, transgene expression levels and molecular profiles were outcrossed and kept for further analysis. The strains used in this study include the following: XQ350 *unc-119(ed3)*; ttTi5605mosII; *xqIs350*(unc-47p::*ATXN3-CAG10*; *unc-119(+)*), and XQ351 *unc-119(ed3)*; ttTi5605mosII; *xqIs351*(unc-47p::*ATXN3-CAG89*; *unc-119(+)*).

### Age-synchronized populations

To obtain an age-synchronized population of worms, ∼8-10 adult hermaphrodites were placed on 10 NGM plates for 3-4 days and kept at 20°C. Then, once the plates contained a large number of adult worms, they were collected with M9 buffer (1 M KH_2_PO_4_, 1 M Na_2_HPO_4_, 1 M NaCl and 1 M MgSO_4_) and centrifuged at 3,250 ***g*** (A-4-81 Rotor) for 4 min at 4°C. After centrifugation, 3 ml of the supernatant was taken and replaced with 3 ml of a mix solution containing NaOH 5 M and bleach (1:2). This was vortexed for 10 min with a high intensity to degrade the worms, leaving a pellet containing only eggs. The pellet was washed four times with M9 buffer and centrifuged at 3,250 ***g*** (A-4-81 Rotor) for 4 min at 4°C. The pellet was transferred onto NGM plates without bacteria and kept overnight at 20°C. The next day, L1 worms were transferred using M9 buffer onto plates streaked with OP50 *E. coli* and kept at 20°C.

### Paralysis assays on solid media

Worms expressing *ATXN3-CAG10*, *ATXN3-CAG89*, *unc-47*p::mCherry; *ATXN3-CAG10* and *unc-47*p::mCherry; *ATXN3-CAG89* were scored for paralysis from adult day 1 to adult day 12. Briefly, 30-40 L4 worms (obtained via synchronization) were transferred onto NGM plates and from adult day 1 to adult day 12, were scored as paralysed if they failed to move after being prodded with a worm pick. Worms were scored as dead if they were unable to respond to tactile head stimulus. They were transferred to fresh plates every 2 days until the cessation of progeny production. All experiments were conducted at 20°C, and each condition was done in triplicates with 30-40 worms per plate.

### Lifespan assays

Approximately 40 L4 worms (obtained via synchronization) were transferred using M9 buffer on new NGM plates and tested daily from adult day 1 until death. Worms were transferred to fresh plates every 2 days until the cessation of progeny production. Worms were scored as dead if they failed to respond to tactile stimulus and showed no spontaneous movement or response when prodded. Dead worms displaying internally hatched progeny or extruded gonads or worms that crawled off the plate were excluded. The transgenic *ATXN3* strains were compared with wild-type worms (N2). All experiments were conducted at 20°C, and each condition was done in triplicates with 40 worms per plate.

### Aldicarb tests

To evaluate synaptic transmission, synchronized worms were grown on NGM plates until adult day 1, 5 and 9. They were then transferred onto NGM plates plus 1 mM aldicarb. Paralysis was scored every 30 min for 2 h on aldicarb plates. Worms were counted as paralysed if they failed to move upon prodding with a worm pick. All experiments were conducted at 20°C. For each strain, the test was done in triplicates with 30 worms per plate in three independent replicates.

### Tracking the movement on solid media

Synchronized adult day 1, 5 and 9 worms were placed on NGM plates for 10 min. Their movement tracking was filmed and photographed using a 12MP Camera installed on a standard laboratory microscope.

### RNAi experiments

Synchronized L1 worms were transferred onto NGM plates enriched with 1 mM isopropyl-β-D-thiogalactopyranoside (IPTG). RNAi-treated *ATXN3-CAG89* worms were fed with *E.coli* containing an empty vector (EV) or expressing dsRNA against *ero-1* (Y105E8B.8) or *ire-1* (C41C4.4). The RNAi clone was obtained from the ORFeome RNAi library (Open Biosystems) and sequence verified. For *ero-1* experiments, age-synchronized worms at adult day 2 were incubated on a slide with 5 µM 2′,7′-dichlorofluorescein diacetate (DCF-DA; Sigma-Aldrich) for 30 min at room temperature and washed three times for 5-10 min with PBS 1× (10 mM Na_2_HPO_4_, 1.8 mM NaH_2_PO_4_ and 140 mM NaCl, adjusted to pH to 7.4; adding ddH_2_O to 1000 ml). Worms were visualized by fluorescence microscopy under 488 nm wavelength excitation.

For *ire-1* experiments, worms were scored from adult day 1 to adult day 12 for paralysis assays. They were transferred to fresh plates every 2 days until the cessation of progeny production. Each condition was done in triplicates with 30-40 worms per plate. All experiments were conducted at 20°C.

### Fluorescence microscopy (neurodegeneration, ER stress response and oxidative stress assays)

For scoring of neuronal processes for gaps or breakages, *unc-47*::mCherry; *ATXN3-CAG89*, *unc-47*::mCherry; *ATXN3-CAG10* and *unc-47*::mCherry were collected at adult day 5 for visualization of motoneuron processes *in vivo.* For visualization of the ER stress response, *hsp-4*::GFP, *hsp-*4::GFP; *ATXN3-CAG10* and *hsp-*4::GFP; *ATXN3-CAG89* worms were collected at adult day 1, and for DCF-DA experiments, wild-type N2 worms and *ATXN3* transgenic lines were collected at adult day 2. The nematodes were immobilized using M9 buffer with 60% glycerol and mounted on slides with 2% agarose pads. mCherry was visualized at 595 nm, and GFP was visualized at 488 nm using a Zeiss Axio Imager M2 microscope. Fluorescent expression was visualized with a DIC microscope Zeiss AxioObserver A1. The software used was AxioVs40 4.8.2.0. One hundred worms were scored per condition for the neurodegeneration assays. Approximately 25 worms were visualized per condition for the ER stress response and the oxidative stress experiments. Image processing and quantification were done with Adobe Photoshop. To compare fluorescence in ER stress response and oxidative stress assays, we calculated the changes in the ratio (size/intensity of fluorescence). Student's unpaired *t-*test was used for statistical analysis.

### Compound testing on solid media

Worms were exposed from hatching (by synchronization) to 60 µM Methylene Blue, 50 µM salubrinal or 50 µM guanabenz incorporated into NGM solid medium, or to NGM solid medium only as a control. All the plates were streaked with OP50 *E. coli.* Compounds were purchased from Sigma-Aldrich (St Louis, MO, USA) and Tocris Bioscience (Ellisville, MO, USA). Briefly, 30-40 worms were picked and plated on the corresponding NGM medium (30-40 worms per plate for each condition, and each condition was done in triplicates) in order to complete the paralysis and lifespan assays, the neurodegeneration observations (fluorescence microscopy) and the drug screens.

### Compound testing in liquid culture

The swimming activity of the nematodes was measured by a WMicroTracker machine (Phylum Tech) (Simonetta and Golombek, 2007). Briefly, worms were exposed until day 5 of adulthood on the corresponding NGM medium plates (drug exposure on solid media) and then were transferred into a 96-well plate. Each well contained a final volume of 100 µl of the drug with the appropriate concentration or M9 buffer used as control, and ∼30 worms adult day 5 (obtained via synchronization). Each condition was done in triplicates, and the experiments were repeated at least three times. The swimming movements of the nematodes were tracked for 10 h.

### Reactive oxygen species measurements

The *in vivo* detection of reactive oxygen species in *C. elegans* has been described previously (Vaccaro et al., 2013, 2012a). Briefly, age-synchronized worms at adult day 2 were incubated on a slide with 5 µM DCF-DA (Sigma-Aldrich) for 30 min at room temperature and washed three times for 5-10 min with PBS 1× (10 mM Na_2_HPO_4_, 1.8 mM NaH_2_PO_4_ and 140 mM NaCl, with pH adjusted to 7.4; adding ddH_2_O to 1000 ml). Worms were visualized by fluorescence microscopy under a 488 nm wavelength excitation.

### Immunofluorescence (antibody staining for transgenic worms)

Five plates of worms for each strain were collected with M9, centrifuged at 3,250 ***g*** (A-4-81 Rotor) for 4 min at 4°C and washed twice with M9 buffer with the same centrifugation conditions. Worm pellets were placed at −80°C overnight. The supernatants were discarded, and 500 µl of cold methanol (stored at −20°C for 5 min) was added to the pellets and remained for 5 min at room temperature (fixation step). The supernatants were discarded, and the pellets were washed twice with PBS 1× (10 mM Na_2_HPO_4_, 1.8 mM NaH_2_PO_4_ and 140 mM NaCl, with pH adjusted to 7.4; adding ddH_2_O to 1000 ml) and centrifuged at 2,151 ***g*** (FA-45-30-11 Rotor) for 3 min at 23°C. The pellets were then blocked with 300 µl donkey serum solution [0.05 mg/l BSA, 5% donkey serum (Sigma-Aldrich; cat. #: D9663; 10 ml), 0.2% Triton] for 30 min at room temperature. Worms were centrifuged at 2,151 ***g*** (FA-45-30-11 Rotor) for 3 min at 23°C and the supernatants discarded. Four hundred microlitres of the primary antibody rabbit anti-ATXN3 (1:200; Proteintech; cat. #: 13505-1) in the donkey serum solution was added to the pellets and stored at 4°C overnight. Worms were washed twice with PBS 1× for 5 min and each time centrifuged at 2,151 ***g*** (FA-45-30-11; Rotor) for 1 min at 23°C to discard the supernatants. The supernatants were discarded, and 400 µl of the secondary antibody donkey anti-rabbit IgG (H+L) Alexa Fluor 488 (1:250; Invitrogen; cat. #:A-21206) in PBS 1× was added to the pellets and remained for 30 min at room temperature. Worms were washed with PBS 1×, centrifuged at 2,151 ***g*** (FA-45-30-11 Rotor) for 1 min at room temperature and the supernatants discarded. Pellets were washed with 400 µl of TOPRO-3 Iodide dye (1:300; Invitrogen; cat. #: T3605) in PBS 1× for 5 min, centrifuged at 2,151 ***g*** (FA-45-30-11 Rotor) for 1 min at 23°C, then washed twice with PBS-T 1× (add 1 ml of Tween to PBS 1× stock) for 5 min at room temperature. Worms were mounted on slides with 20 µl mounting solution (Invitrogen ProLong Antifade Kit; cat. #: P7481) and stored at 4°C overnight. Confocal images were acquired on a Leica TCS-SP5 inverted confocal microscope using an HCX PL APO CS 40×/1.25 oil objective. Excitation system was performed using a 633 HeNe laser for TOPRO simultaneously with the 488 nm line of an argon laser for eGFP. Scan speed was 400 Hz. Detection bandwidth was 643-800 nm for TOPRO-3, and 498-551 nm for eGFP. The software used was LAS Image Analysis. Twenty-five to thirty worms were visualized per condition for the aggregation phenotype. Image processing was done with LAS Image Analysis and Adobe Photoshop.

### Cell lysis

Cells derived from healthy and MJD patients were obtained from Dr Guy Rouleau (Montreal Neurological Institute and Hospital, McGill University). MJD lymphoblastoid cell lines (LCL) were established from peripheral blood samples of MJD patients of European origin. Cells were grown in IMDM (Gibco) supplemented with fetal bovine serum (10%), penicillin and streptomycin (100 units/ml) and L-glutamine (0.292 mg/ml). Collected cells were centrifuged at 98 ***g*** (FA-45-24-11 Rotor) for 2 min at 4°C. The supernatants were discarded and the pellets transferred into new Eppendorf tubes with 1 ml cold PBS 1× (10 mM Na_2_HPO_4_, 1.8 mM NaH_2_PO_4_ and 140 mM NaCl, with pH adjusted to 7.4; adding ddH_2_O to 1000 ml) and centrifuged at 98 ***g*** for 5 min at 4°C. This step was repeated three times. The medium was aspirated and the pellets were resuspended in an appropriate volume of RIPA buffer (150 mM NaCl, 50 mM Tris pH 7.4, 1% Triton X-100, 0.1% SDS and 1% sodium deoxycholate) and 0.1% protease inhibitors (10 mg/ml leupeptin, 10 mg/ml pepstatin A and 10 mg/ml chymostatin LPC; 1/1000) depending on pellet size. The samples were put on ice for 10 min, then placed at room temperature for 10 min. The samples were then centrifuged at 142 ***g*** (FA-45-24-11 Rotor) for 15 min at 4°C. The pellets were discarded, and the supernatants were collected in 1.5 ml tubes and stored at −80°C.

### Western blotting

Fifteen plates of worms for each strain and for each condition (with or without compounds) were collected with M9, centrifuged at 3,250 ***g*** (A-4-81 Rotor) for 4 min at 4°C and washed twice with M9 buffer with the same centrifugation conditions. Worm pellets were placed at −80°C overnight. Pellets were lysed in RIPA buffer (150 mM NaCl, 50 mM Tris pH 7.4, 1% Triton X-100, 0.1% SDS and 1% sodium deoxycholate) and 0.1% protease inhibitors (10 mg/ml leupeptin, 10 mg/ml pepstatin A and 10 mg/ml chymostatin LPC; 1/1000). Pellets were passed through a 27.5-gauge syringe seven times, sonicated for 5 min, and centrifuged at 16,000 ***g*** for 15 min at 4°C. Supernatants were collected in 1.5 ml tubes. The supernatants were quantified using the BCA protein assay kit (Thermo Scientific) following the manufacturer's protocol and instructions.

Thirty-five micrograms per well of protein were loaded in a 10% polyacrylamide gel for 80 min, transferred to nitrocellulose membranes (BioRad) and immunoblotted. Antibodies used were as follows: rabbit anti-ATXN3 (1:500; Proteintech; cat. #: 13505-1) and mouse anti-Actin (1:2500; MP Biomedical; cat. #: 691001). Blots were visualized using peroxidase-conjugated secondary antibodies and ECL western blotting substrate (Thermo Scientific). N2 wild-type worms and *atx-3(tm1689)* were used as controls. The ladder used was Precision Plus Protein Kaleidoscope (BioRad). Densitometry was performed with Adobe Photoshop.

### Protein solubility

Fifteen plates of worms for each strain and for each condition (*ATXN-CAG89* worms treated with each compound) were collected with M9, centrifuged at 3,250 ***g*** (A-4-81 Rotor) for 4 min at 4°C and washed twice with M9 buffer with the same centrifugation conditions. Worm pellets were placed at −80°C overnight. To obtain soluble and insoluble fractions for our transgenics, worms were lysed in extraction buffer (1 M Tris-HCl pH 8, 0.5 M EDTA, 1 M NaCl and 10% SDS) plus protease inhibitors (10 mg/ml leupeptin, 10 mg/ml pepstatin A and 10 mg/ml chymostatin LPC; 1/1000). Pellets were passed through a 27.5-gauge syringe 10 times, sonicated for 5 min, and centrifuged at 16,000 ***g*** for 15 min at 4°C. The soluble supernatants were collected in 1.5 ml tubes and stored at −80°C. The remaining pellets were resuspended in extraction buffer, sonicated and centrifuged at 10,000 ***g*** for 5 min. The supernatants were discarded and the remaining pellets resuspended in 100 μl of RIPA buffer and sonicated for ∼90 min until the pellets were resuspended in solution. The pellets were collected and stored in 1.5 ml tubes at −80°C. The supernatants and pellets were quantified using the BCA protein assay kit (Thermo Scientific) following the manufacturer's protocol and instructions.

### Dot blotting

Dot blotting was done using the Bio-Dot SF microfiltration apparatus (cat. #: 170-6542). The assays were done using the manufacturer's protocol and instructions following the section Protein Slot Blotting-Immunoassays procedure and followed immunoblotting procedures. The antibody used was rabbit anti-ATXN3 (1:500; Proteintech; cat. #: 13505-1). Blots were visualized using peroxidase-conjugated secondary antibodies and ECL western blotting substrate (Thermo Scientific) via the Bio Rad ChemiDoc MP Imaging System (model #: Universal HOOD III). N2 wild-type worms and *ATXN3-CAG10* were used as controls. Quantification was performed with Image Lab software.

### Statistics

Paralysis and lifespan curves were generated and compared using the log-rank (Mantel–Cox) test. All experiments were repeated at least three times. For neurodegeneration, the drug screening and the fluorescence tests (oxidative stress and ER stress response), Student's unpaired *t-*tests were performed. For the dot blotting quantification, ANOVAs were used. Prism 6 (GraphPad Software) was used for all statistical analyses.

## Supplementary Material

Supplementary information

## References

[DMM029736C1] Bettencourt C., Santos C., Kay T., Vasconcelos J. and Lima M. (2008). Analysis of segregation patterns in Machado-Joseph disease pedigrees. *J. Hum. Genet.* 53, 920-923. 10.1007/s10038-008-0330-y 18688568

[DMM029736C2] Bonanomi M., Natalello A., Visentin C., Pastori V., Penco A., Cornelli G., Colombo G., Malabarba M. G., Doglia S. M., Relini A. et al. (2014). Epigallocatechin-3-gallate and tetracycline differently affect ataxin-3 fibrillogenesis and reduce toxicity in spinocerebellar ataxia type 3 model. *Hum. Mol. Genet.* 23, 6542-6552. 10.1093/hmg/ddu373 25030034

[DMM029736C3] Breuer P., Haacke A., Evert B. O. and Wüllner U. (2010). Nuclear aggregation of polyglutamine-expanded ataxin-3: fragments escape the cytoplasmic quality control. *J. Biol. Chem.* 285, 6532-6537. 10.1074/jbc.M109.036335 20064935 PMC2825449

[DMM029736C4] C. elegans Deletion Mutant Consortium. (2012). large-scale screening for targeted knockouts in the Caenorhabditis elegans genome. *G3 (Bethesda).* 2, 1415-1425. 10.1534/g3.112.003830 23173093 PMC3484672

[DMM029736C5] Catoire H., Pasco M. Y., Abu-Baker A., Holbert S., Tourette C., Brais B., Rouleau G. A., Parker J. A. and Néri C. (2008). Sirtuin inhibition protects from the polyalanine muscular dystrophy protein PABPN1. *Hum. Mol. Genet.* 17, 2108-2117. 10.1093/hmg/ddn109 18397876

[DMM029736C6] Christie N. T. M., Lee A. L., Fay H. G., Gray A. A. and Kikis E. A. (2014). Novel polyglutamine model uncouples proteotoxicity from aging. *PLoS ONE* 9, e96835 10.1371/journal.pone.0096835 24817148 PMC4016013

[DMM029736C7] Collins J. J., Huang C., Hughes S. and Kornfeld K. (2008). The measurement and analysis of age-related changes in Caenorhabditis elegans. *WormBook*, 1-21. 10.1895/wormbook.1.137.1 PMC4781475 18381800

[DMM029736C8] Costa M. C. and Paulson H. L. (2012). Toward understanding Machado-Joseph disease. *Prog. Neurobiol.* 97, 239-257. 10.1016/j.pneurobio.2011.11.006 22133674 PMC3306771

[DMM029736C9] Cummings C. J. and Zoghbi H. Y. (2000). Fourteen and counting: unraveling trinucleotide repeat diseases. *Hum. Mol. Genet.* 9, 909-916. 10.1093/hmg/9.6.909 10767314

[DMM029736C10] Echtermeyer C., da Fontoura Costa L., Rodrigues F. A. and Kaiser M. (2011). Automatic network fingerprinting through single-node motifs. *PLoS ONE* 6, e15765 10.1371/journal.pone.0015765 21297963 PMC3031529

[DMM029736C11] Evers M. M., Toonen L. J. and van Roon-Mom W. M. (2014). Ataxin-3 protein and RNA toxicity in spinocerebellar ataxia type 3: current insights and emerging therapeutic strategies. *Mol. Neurobiol.* 49, 1513-1531. 10.1007/s12035-013-8596-2 24293103 PMC4012159

[DMM029736C12] França M. C. Jr, D'Abreu A., Nucci A. and Lopes-Cendes I. (2008). Muscle excitability abnormalities in Machado-Joseph disease. *Arch. Neurol.* 65, 525-529. 10.1001/archneur.65.4.525 18413477

[DMM029736C13] Frokjaer-Jensen C., Davis M. W., Sarov M., Taylor J., Flibotte S., LaBella M., Pozniakovsky A., Moerman D. G. and Jorgensen E. M. (2014). Random and targeted transgene insertion in Caenorhabditis elegans using a modified Mos1 transposon. *Nat. Methods* 11, 529-534. 10.1038/nmeth.2889 24820376 PMC4126194

[DMM029736C14] Gatchel J. R. and Zoghbi H. Y. (2005). Diseases of unstable repeat expansion: mechanisms and common principles. *Nat. Rev. Genet.* 6, 743-755. 10.1038/nrg1691 16205714

[DMM029736C15] Goto J., Watanabe M., Ichikawa Y., Yee S.-B., Ihara N., Endo K., Igarashi S., Takiyama Y., Gaspar C., Maciel P. et al. (1997). Machado-Joseph disease gene products carrying different carboxyl termini. *Neurosci. Res.* 28, 373-377. 10.1016/S0168-0102(97)00056-4 9274833

[DMM029736C16] Harding H. P., Zhang Y., Zeng H., Novoa I., Lu P. D., Calfon M., Sadri N., Yun C., Popko B., Paules R. et al. (2003). An integrated stress response regulates amino acid metabolism and resistance to oxidative stress. *Mol. Cell* 11, 619-633. 10.1016/S1097-2765(03)00105-9 12667446

[DMM029736C17] Herndon L. A., Schmeissner P. J., Dudaronek J. M., Brown P. A., Listner K. M., Sakano Y., Paupard M. C., Hall D. H. and Driscoll M. (2002). Stochastic and genetic factors influence tissue-specific decline in ageing C. elegans. *Nature* 419, 808-814. 10.1038/nature01135 12397350

[DMM029736C18] Julien C., Lissouba A., Madabattula S., Fardghassemi Y., Rosenfelt C., Androschuk A., Strautman J., Wong C., Bysice A., O'Sullivan J. et al. (2016). Conserved pharmacological rescue of hereditary spastic paraplegia-related phenotypes across model organisms. *Hum. Mol. Genet.* 25, 1088-1099. 10.1093/hmg/ddv632 26744324 PMC4764191

[DMM029736C19] Kanai K. and Kuwabara S. (2009). Motor nerve hyperexcitability and muscle cramps in Machado-Joseph disease. *Arch. Neurol.* 66, 139; author reply 139-140 10.1001/archneurol.2008.515 19139316

[DMM029736C20] Kawaguchi Y., Okamoto T., Taniwaki M., Aizawa M., Inoue M., Katayama S., Kawakami H., Nakamura S., Nishimura M., Akiguchi I. et al. (1994). CAG expansions in a novel gene for Machado-Joseph disease at chromosome 14q32.1. *Nat. Genet.* 8, 221-228. 10.1038/ng1194-221 7874163

[DMM029736C21] Khan L. A., Bauer P. O., Miyazaki H., Lindenberg K. S., Landwehrmeyer B. G. and Nukina N. (2006). Expanded polyglutamines impair synaptic transmission and ubiquitin-proteasome system in Caenorhabditis elegans. *J. Neurochem.* 98, 576-587. 10.1111/j.1471-4159.2006.03895.x 16805848

[DMM029736C22] Koch P., Breuer P., Peitz M., Jungverdorben J., Kesavan J., Poppe D., Doerr J., Ladewig J., Mertens J., Tuting T. et al. (2011). Excitation-induced ataxin-3 aggregation in neurons from patients with Machado-Joseph disease. *Nature* 480, 543-546. 10.1038/nature10671 22113611

[DMM029736C23] Li X., Liu H., Fischhaber P. L. and Tang T.-S. (2015). Toward therapeutic targets for SCA3: insight into the role of Machado-Joseph disease protein ataxin-3 in misfolded proteins clearance. *Prog. Neurobiol.* 132, 34-58. 10.1016/j.pneurobio.2015.06.004 26123252

[DMM029736C24] Loria P. M., Hodgkin J. and Hobert O. (2004). A conserved postsynaptic transmembrane protein affecting neuromuscular signaling in Caenorhabditis elegans. *J. Neurosci.* 24, 2191-2201. 10.1523/JNEUROSCI.5462-03.2004 14999070 PMC6730426

[DMM029736C25] Maciel P., Costa M. C., Ferro A., Rousseau M., Santos C. S., Gaspar C., Barros J., Rouleau G. A., Coutinho P. and Sequeiros J. (2001). Improvement in the molecular diagnosis of Machado-Joseph disease. *Arch. Neurol.* 58, 1821-1827. 10.1001/archneur.58.11.1821 11708990

[DMM029736C26] Mahoney T. R., Luo S. and Nonet M. L. (2006). Analysis of synaptic transmission in Caenorhabditis elegans using an aldicarb-sensitivity assay. *Nat. Protoc.* 1, 1772-1777. 10.1038/nprot.2006.281 17487159

[DMM029736C27] Matos C. A., de Macedo-Ribeiro S. and Carvalho A. L. (2011). Polyglutamine diseases: the special case of ataxin-3 and Machado-Joseph disease. *Prog. Neurobiol.* 95, 26-48. 10.1016/j.pneurobio.2011.06.007 21740957

[DMM029736C28] McIntire S. L., Jorgensen E., Kaplan J. and Horvitz H. R. (1993). The GABAergic nervous system of Caenorhabditis elegans. *Nature* 364, 337-341. 10.1038/364337a0 8332191

[DMM029736C29] McIntire S. L., Reimer R. J., Schuske K., Edwards R. H. and Jorgensen E. M. (1997). Identification and characterization of the vesicular GABA transporter. *Nature.* 389, 870-876. 10.1038/39908 9349821

[DMM029736C30] Petrash H. A., Philbrook A., Haburcak M., Barbagallo B. and Francis M. M. (2013). ACR-12 ionotropic acetylcholine receptor complexes regulate inhibitory motor neuron activity in Caenorhabditis elegans. *J. Neurosci.* 33, 5524-5532. 10.1523/JNEUROSCI.4384-12.2013 23536067 PMC3645261

[DMM029736C31] Piano F., Schetter A. J., Morton D. G., Gunsalus K. C., Reinke V., Kim S. K. and Kemphues K. J. (2002). Gene clustering based on RNAi phenotypes of ovary-enriched genes in C. elegans. *Curr. Biol.* 12, 1959-1964. 10.1016/S0960-9822(02)01301-5 12445391

[DMM029736C32] Pinto S. and De Carvalho M. (2008). Machado-Joseph disease presenting as motor neuron disease. *Amyotroph. Lateral. Scler.* 9, 188-191. 10.1080/17482960701702603 17963092

[DMM029736C33] Reina C. P., Zhong X. and Pittman R. N. (2010). Proteotoxic stress increases nuclear localization of ataxin-3. *Hum. Mol. Genet.* 19, 235-249. 10.1093/hmg/ddp482 19843543 PMC2796889

[DMM029736C34] Riess O., Rüb U., Pastore A., Bauer P. and Schöls L. (2008). SCA3: neurological features, pathogenesis and animal models. *Cerebellum* 7, 125-137. 10.1007/s12311-008-0013-4 18418689

[DMM029736C35] Rodrigues A. J., Coppola G., Santos C., Costa Mdo C., Ailion M., Sequeiros J., Geschwind D. H. and Maciel P. (2007). Functional genomics and biochemical characterization of the C. elegans orthologue of the Machado-Joseph disease protein ataxin-3. *FASEB J.* 21, 1126-1136. 10.1096/fj.06-7002com 17234717

[DMM029736C36] Rodrigues A. J., Neves-Carvalho A., Teixeira-Castro A., Rokka A., Corthals G., Logarinho E. and Maciel P. (2011). Absence of ataxin-3 leads to enhanced stress response in C. elegans. *PLoS ONE* 6, e18512 10.1371/journal.pone.0018512 21526185 PMC3079722

[DMM029736C37] Rub U., de Vos R. A. I., Schultz C., Brunt E. R., Paulson H. and Braak H. (2002). Spinocerebellar ataxia type 3 (Machado-Joseph disease): severe destruction of the lateral reticular nucleus. *Brain* 125, 2115-2124. 10.1093/brain/awf208 12183356

[DMM029736C38] Rub U., Brunt E. R. and Deller T. (2008). New insights into the pathoanatomy of spinocerebellar ataxia type 3 (Machado-Joseph disease). *Curr. Opin. Neurol.* 21, 111-116. 10.1097/WCO.0b013e3282f7673d 18317266

[DMM029736C39] Saxena S. and Caroni P. (2011). Selective neuronal vulnerability in neurodegenerative diseases: from stressor thresholds to degeneration. *Neuron* 71, 35-48. 10.1016/j.neuron.2011.06.031 21745636

[DMM029736C40] Schmeisser K., Fardghassemi Y. and Parker J. A. (2017). A rapid chemical-genetic screen utilizing impaired movement phenotypes in C. elegans: Input into genetics of neurodevelopmental disorders. *Exp. Neurol.* 293, 101-114. 10.1016/j.expneurol.2017.03.022 28373024

[DMM029736C41] Schöls L., Bauer P., Schmidt T., Schulte T. and Riess O. (2004). Autosomal dominant cerebellar ataxias: clinical features, genetics, and pathogenesis. *Lancet. Neurol.* 3, 291-304. 10.1016/S1474-4422(04)00737-9 15099544

[DMM029736C42] Shao J. and Diamond M. I. (2007). Polyglutamine diseases: emerging concepts in pathogenesis and therapy. *Hum. Mol. Genet.* 16, R115-R123. 10.1093/hmg/ddm213 17911155

[DMM029736C43] Simonetta S. H. and Golombek D. A. (2007). An automated tracking system for Caenorhabditis elegans locomotor behavior and circadian studies application. *J. Neurosci. Methods* 161, 273-280. 10.1016/j.jneumeth.2006.11.015 17207862

[DMM029736C44] Soon B.-W., Cheng C.-H., Liu R.-S. and Shan D.-E. (1997). Machado-Joseph disease: clinical, molecular, and metabolic characterization in Chinese kindreds. *Ann. Neurol.* 41, 446-452. 10.1002/ana.410410407 9124801

[DMM029736C45] Stiernagle T. (2006). Maintenance of C. elegans. *WormBook*, 1-11. 10.1895/wormbook.1.101.1 PMC4781397 18050451

[DMM029736C46] Stochmanski S. J., Therrien M., Laganière J., Rochefort D., Laurent S., Karemera L., Gaudet R., Vyboh K., Van Meyel D. J., Di Cristo G. et al. (2012). Expanded ATXN3 frameshifting events are toxic in Drosophila and mammalian neuron models. *Hum. Mol. Genet.* 21, 2211-2218. 10.1093/hmg/dds036 22337953

[DMM029736C47] Takiyama Y., Oyanagi S., Kawashima S., Sakamoto H., Saito K., Yoshida M., Tsuji S., Mizuno Y. and Nishizawa M. (1994). A clinical and pathologic study of a large Japanese family with Machado- Joseph disease tightly linked to the DNA markers on chromosome 14q. *Neurology* 44, 1302-1308. 10.1212/WNL.44.7.1302 8035935

[DMM029736C48] Taylor R. C. and Dillin A. (2013). XBP-1 is a cell-nonautonomous regulator of stress resistance and longevity. *Cell* 153, 1435-1447. 10.1016/j.cell.2013.05.042 23791175 PMC4771415

[DMM029736C49] Teixeira-Castro A., Ailion M., Jalles A., Brignull H. R., Vilaça J. L., Dias N., Rodrigues P., Oliveira J. F., Neves-Carvalho A., Morimoto R. I. et al. (2011). Neuron-specific proteotoxicity of mutant ataxin-3 in C. elegans: rescue by the DAF-16 and HSF-1 pathways. *Hum. Mol. Genet.* 20, 2996-3009. 10.1093/hmg/ddr203 21546381 PMC3131043

[DMM029736C50] Teixeira-Castro A., Jalles A., Esteves S., Kang S., da Silva Santos L., Silva-Fernandes A., Neto M. F., Brielmann R. M., Bessa C., Duarte-Silva S. et al. (2015). Serotonergic signalling suppresses ataxin 3 aggregation and neurotoxicity in animal models of Machado-Joseph disease. *Brain* 138, 3221-3237. 10.1093/brain/awv262 26373603 PMC4731417

[DMM029736C51] Therrien M. and Parker J. A. (2014). Worming forward: amyotrophic lateral sclerosis toxicity mechanisms and genetic interactions in Caenorhabditis elegans. *Front. Genet.* 5, 85 10.3389/fgene.2014.00085 24860590 PMC4029022

[DMM029736C52] Urano F., Calfon M., Yoneda T., Yun C., Kiraly M., Clark S. G. and Ron D. (2002). A survival pathway for Caenorhabditis elegans with a blocked unfolded protein response. *J. Cell Biol.* 158, 639-646. 10.1083/jcb.200203086 12186849 PMC2174003

[DMM029736C53] Vaccaro A., Patten S. A., Ciura S., Maios C., Therrien M., Drapeau P., Kabashi E. and Parker J. A. (2012a). Methylene blue protects against TDP-43 and FUS neuronal toxicity in C. elegans and D. rerio. *PLoS ONE* 7, e42117 10.1371/journal.pone.0042117 22848727 PMC3407135

[DMM029736C54] Vaccaro A., Tauffenberger A., Aggad D., Rouleau G., Drapeau P. and Parker J. A. (2012b). Mutant TDP-43 and FUS cause age-dependent paralysis and neurodegeneration in C. elegans. *PLoS ONE* 7, e31321 10.1371/journal.pone.0031321 22363618 PMC3283630

[DMM029736C55] Vaccaro A., Tauffenberger A., Ash P. E. A., Carlomagno Y., Petrucelli L. and Parker J. A. (2012c). TDP-1/TDP-43 regulates stress signaling and age-dependent proteotoxicity in Caenorhabditis elegans. *PLoS Genet.* 8, e1002806 10.1371/journal.pgen.1002806 22792076 PMC3390363

[DMM029736C56] Vaccaro A., Patten S. A., Aggad D., Julien C., Maios C., Kabashi E., Drapeau P. and Parker J. A. (2013). Pharmacological reduction of ER stress protects against TDP-43 neuronal toxicity in vivo. *Neurobiol. Dis.* 55, 64-75. 10.1016/j.nbd.2013.03.015 23567652

[DMM029736C57] van de Warrenburg B. P. C., Sinke R. J., Verschuuren-Bemelmans C. C., Scheffer H., Brunt E. R., Ippel P. F., Maat-Kievit J. A., Dooijes D., Notermans N. C., Lindhout D. et al. (2002). Spinocerebellar ataxias in the Netherlands: prevalence and age at onset variance analysis. *Neurology* 58, 702-708. 10.1212/WNL.58.5.702 11889231

[DMM029736C58] van Oosten-Hawle P., Porter R. S. and Morimoto R. I. (2013). Regulation of organismal proteostasis by transcellular chaperone signaling. *Cell* 153, 1366-1378. 10.1016/j.cell.2013.05.015 23746847 PMC3955170

[DMM029736C59] Veriepe J., Fossouo L. and Parker J. A. (2015). Neurodegeneration in C. elegans models of ALS requires TIR-1/Sarm1 immune pathway activation in neurons. *Nat. Commun.* 6, 7319 10.1038/ncomms8319 26059317

[DMM029736C60] Walker A. K. and Atkin J. D. (2011). Stress signaling from the endoplasmic reticulum: a central player in the pathogenesis of amyotrophic lateral sclerosis. *IUBMB Life* 63, 754-763. 10.1002/iub.520 21834058

[DMM029736C61] Wang J., Farr G. W., Hall D. H., Li F., Furtak K., Dreier L. and Horwich A. L. (2009). An ALS-linked mutant SOD1 produces a locomotor defect associated with aggregation and synaptic dysfunction when expressed in neurons of Caenorhabditis elegans. *PLoS Genet.* 5, e1000350 10.1371/journal.pgen.1000350 19165329 PMC2621352

[DMM029736C62] Wüllner U., Reimold M., Abele M., Bürk K., Minnerop M., Dohmen B.-M., Machulla H.-J., Bares R. and Klockgether T. (2005). Dopamine transporter positron emission tomography in spinocerebellar ataxias type 1, 2, 3, and 6. *Arch. Neurol.* 62, 1280-1285. 10.1001/archneur.62.8.1280 16087769

[DMM029736C63] Xu Z., Joel Tito A., Rui Y.-N. and Zhang S. (2015). Studying polyglutamine diseases in Drosophila. *Exp. Neurol.* 274, 25-41. 10.1016/j.expneurol.2015.08.002 26257024 PMC4644473

[DMM029736C64] Yen T. C., Lu C. S., Tzen K. Y., Wey S. P., Chou Y. H., Weng Y. H., Kao P. F. and Ting G. (2000). Decreased dopamine transporter binding in Machado-Joseph disease. *J. Nucl. Med.* 41, 994-998. 10855623

