# Supplementary material for: Rescue of ATXN3 neuronal toxicity in Caenorhabditis elegans by chemical modification of endoplasmic reticulum stress
Source: Dis Model Mech. 2017 Dec 1;10(12):1465–80. doi: 10.1242/dmm.029736 (PMC5769603; doi:10.1242/dmm.029736)
Supplement: Supplementary information [file dmm-10-029736-s1.pdf]

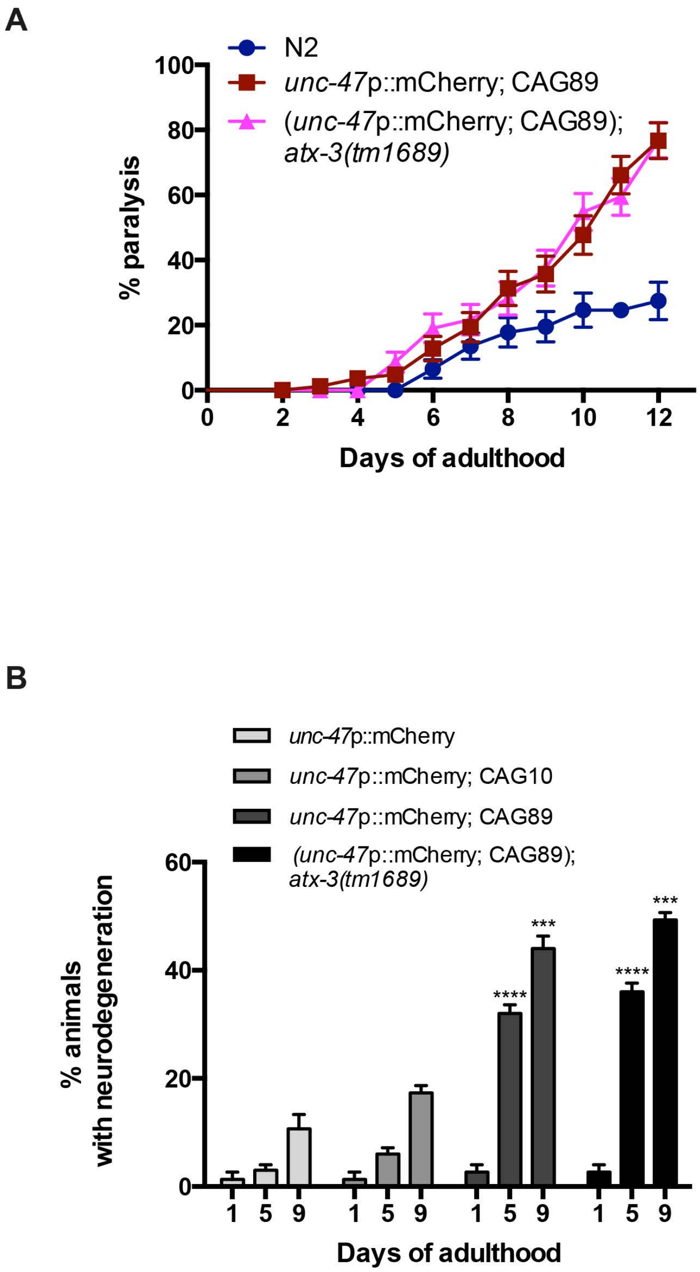


Supplementary Figure 1. atx-3(tm1689) do not affect the paralysis and neurodegeneration phenotypes observed in ATXN3-CAG89 transgenic worms

(A) No difference between unc-47p::mCherry; CAG89 and (unc-47p::mCherry; CAG89); atx-3(tm1689) transgenic worms were identified for the paralysis phenotype. This experiment was replicated for 3 times.

(B) Quantification of neurodegeneration in transgenic worms at days one, five and nine of adulthood. At adult day one worms, there was none significant neurodegeneration phenotype for the transgenic worms when compared to unc-47p::mCherry. ATXN3-CAG89 transgenics showed a significant increase of neurodegeneration compared to unc-47p::mCherry and ATXN3-CAG10 controls (****$P < 0.0001$ for day five of adulthood and ***$P < 0.001$ for adult dav nine worms, by Student's t-test) (N=100 for each condition). No difference between unc-47p::mCherry; CAG89 and (unc-47p::mCherry; CAG89); atx-3(tm1689) transgenic worms were identified for the neurodegeneration phenotype for none of the tree days of the study (days one, five and nine of adulthood). These experiments were replicated for 3 times.

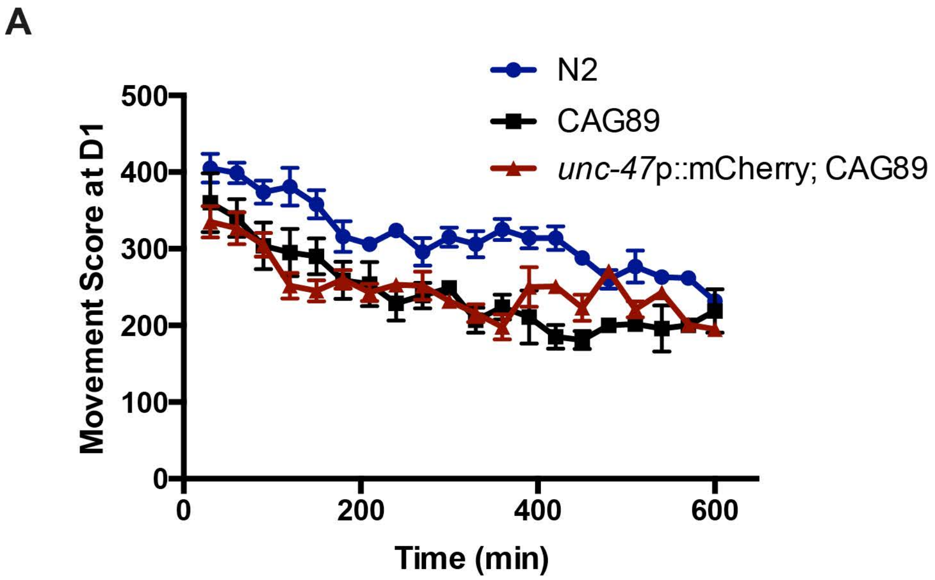


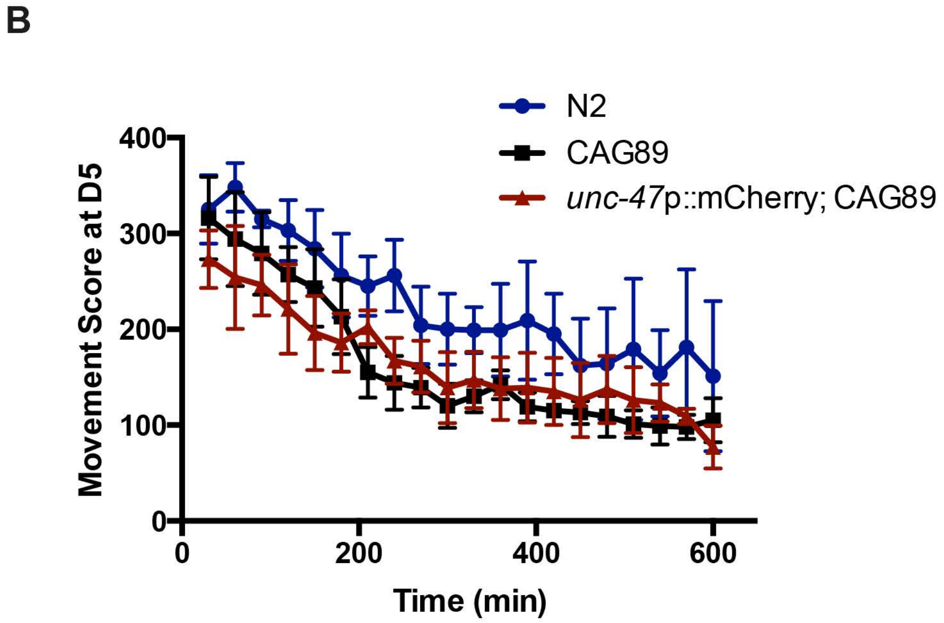


Supplementary Figure 2. unc-47p::mCherry do not affect the swimming activity of ATXN3-CAG89 mutant worms

The swimming activity of ATXN3-CAG89 mutants was compared to unc-47p::mCherry; CAG89 worms for a period of ten hours at (A) day one of adulthood and, (B) day five of adulthood. No differences for the motility was observed between ATXN3-CAG89 and unc-47p::mCherry; CAG89 worms.
